# Evidence for a chemical clock in oscillatory formation of UiO-66

**DOI:** 10.1038/ncomms11832

**Published:** 2016-06-10

**Authors:** M. G. Goesten, M. F. de Lange, A. I. Olivos-Suarez, A. V. Bavykina, P. Serra-Crespo, C. Krywka, F. M. Bickelhaupt, F. Kapteijn, Jorge Gascon

**Affiliations:** 1 Catalysis Engineering, Delft University of Technology, Julianalaan 136, 2628 BL Delft, The Netherlands; 2 Inorganic Materials Chemistry, Eindhoven University of Technology, P.O. Box 513, 5600 MB Eindhoven, The Netherlands; 3 Radiation Science and Technology, Mekelweg 15, 2629 JB Delft, The Netherlands; 4 National Synchrotron Light Source, Brookhaven National Laboratory, Upton, New York 11973, USA; 5 Helmholtz-Zentrum Geesthacht, D-21502 Geesthacht, Germany; 6 Department of Theoretical Chemistry and Amsterdam Center for Multiscale Modeling (ACMM), VU University, de Boelelaan 1083, 1081 HV Amsterdam, The Netherlands; 7 Institute of Molecules and Materials (IMM), Radboud University, Heyendaalseweg 135, 6525 AJ Nijmegen, The Netherlands

## Abstract

Chemical clocks are often used as exciting classroom experiments, where an induction time is followed by rapidly changing colours that expose oscillating concentration patterns. This type of reaction belongs to a class of nonlinear chemical kinetics also linked to chaos, wave propagation and Turing patterns. Despite its vastness in occurrence and applicability, the clock reaction is only well understood for liquid-state processes. Here we report a chemical clock reaction, in which a solidifying entity, metal–organic framework UiO-66, displays oscillations in crystal dimension and number, as shown by X-ray scattering. In rationalizing this result, we introduce a computational approach, the metal–organic molecular orbital methodology, to pinpoint interaction between the tectonic building blocks that construct the metal–organic framework material. In this way, we show that hydrochloric acid plays the role of autocatalyst, bridging separate processes of condensation and crystallization.

## Abstract (web summary)

Reactions with non-linear kinetics, such as chemical clocks, are reasonably common but only well understood in the liquid phase. Here, the authors report and rationalize a chemical clock reaction taking place in a solidifying metal-organic framework.

## Graphical abstract

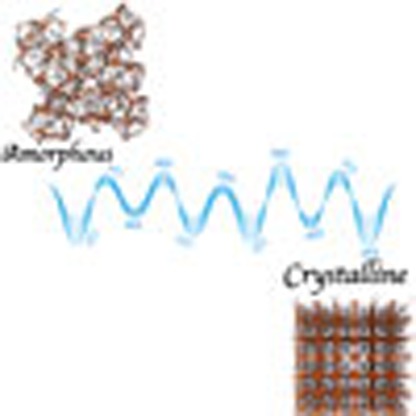

Achemical clock is a particular type of chemical reaction that is frequently applied in chemistry education. The precise definition of a chemical clock is cause for some debate^1^, but it is generally accepted that the reaction possesses an induction time after which a sudden and marked change in concentration of the reactant and product species ensues^2^. A reaction based on hydrogen peroxide, iodide and thiosulfate is popular^3^:


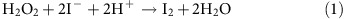



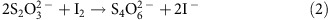


The first reaction is slow, the second one fast. As a result of the difference in reaction rates and the fact concentrations of the autocatalytic redox-pair iodide/iodine are coupled in nonlinear kinetics, the iodine concentration is seen to shoot up after an initial lag time after which it oscillates to equilibrium. Iodide is not consumed in the overall reaction and serves as autocatalyst. If starch is added, the oscillations can be followed by corresponding changes in the solution colour. This makes it a popular classroom reaction. In broad context, clock reactions belong to a class of nonlinear chemical phenomena linked to several natural processes and rhythms. Great interest exists in coupling oscillatory clock reactions to processes occurring in the solid state^4^. A particularly exciting development is the use of chemical actuation to fabricate a synthetic muscle. It is possible to use a clock reaction to produce oscillatory pH or temperature trajectories that drive a muscle-like mechanical response from a polymeric material^5^. For example, Howse *et al*.^6^ fabricated in this way a device capable of producing 20 mW kg^−1^ of power. These are solution-driven oscillations in the solid state, but ‘direct' oscillatory behaviour exists in conversion and temperature during certain polymerization reactions^7^. Clock reactions involving phase transitions, in which for instance precipitating or crystallizing particle dimensions oscillate during the course of reaction, have never been shown to exist, despite nonlinear dynamics suspected to govern several important condensation processes, such as the hardening of cement^8^.

In crystal engineering studies—the design and synthesis of functional crystalline structures based on a bottom-up approach from smaller building blocks—scientists have shown remarkable chemical mechanisms to prelude and govern synthesis of metal–organic framework (MOF) crystals^9^,^10^,^11^,^12^,^13^,^14^,^15^,^16^. MOFs are porous materials consisting of metal clusters that are linked to each other by organic bridging ligands. MOFs carry, arguably more than any other type of material, the potential to fulfil crystal engineering, for the cluster and ligand connectivity, and properties, can be to certain extent controlled^17^. This control is limited, much because of the unpredictability of coordination chemistry around the tectonic building blocks of MOFs^14^,^18^. One example is a report by Farha *et al*. on the synthesis of the ultra-stable and highly popular MOF UiO-66, which is based on hexanuclear zirconium/hafnium oxoclusters, and terephthalate. The authors showed that the addition of hydrochloric acid (HCl) considerably speeds up the synthesis^19^. This is surprising, because HCl is formed during the synthesis, which (based on naive grounds) would suggest that its neutralization, rather than addition would lead to improvement in synthesis by favourably shifting the equilibrium towards the formation of the MOF. The only scenario, in which HCl is formed *in situ*, while being kinetically beneficial matters the scenario of HCl being autocatalytic, fulfilling a similar role to iodide in reactions 1 and 2, and possibly sparking spectacular phenomena during the formation of UiO-66.

Using a combination of *in situ* synchrotron X-ray scattering and a quantum chemical methodology, we herein show that HCl is indeed autocatalytic; as a consequence, the formation of UiO-66 involves a clock reaction with a lag time followed by an oscillatory wave front in crystal dimensions.

## Results

### X-ray scattering

*In situ* small- and wide-angle X-ray scattering (SAXS/WAXS) is well suited to follow crystallization and condensation processes. If synchrotron radiation is used, time-resolved data of high resolution can be obtained on multiple length scales of self-assembly. The small-angle camera follows events on the colloidal (2–50 nm) length scale, permitting time-resolved analysis of the solidifying entity, while the wide-angle camera follows the evolution of Bragg peaks in case of evolution of a crystalline compound^16^. Important, and of great use in this work, is the possibility to deconvolute condensation from crystallization by respectively analysing SAXS versus WAXS data.

The SAXS/WAXS measurements were carried out at the X9 beamline at the National Synchtrotron Light Source (NSLS), Brookhaven, NY, USA. SAXS data were processed to obtain temporal information on the following integral parameters:

·Porod volume, *V*_p_(nm^3^): volume of the particle.

·Radius of gyration *R*_g_ (nm): root mean square average of all distances from the particle boundary to its centre of mass.

·Number densiy *N/V* (−): the number of particles per unit volume, in arbitrary units. Only the trend is followed.

·Porod surface-to-volume *S/V* (nm^−1^): specific surface area of the precipitating particle.

·During the experiments, WAXS was used to follow:

·Extent of crystallization *α* (−): extent parameter obtained via Bragg peak evolution. In this case focused on the most dominant reflection of UiO-66 at *q*=0.5 nm^−1^.

**Figure 1:**
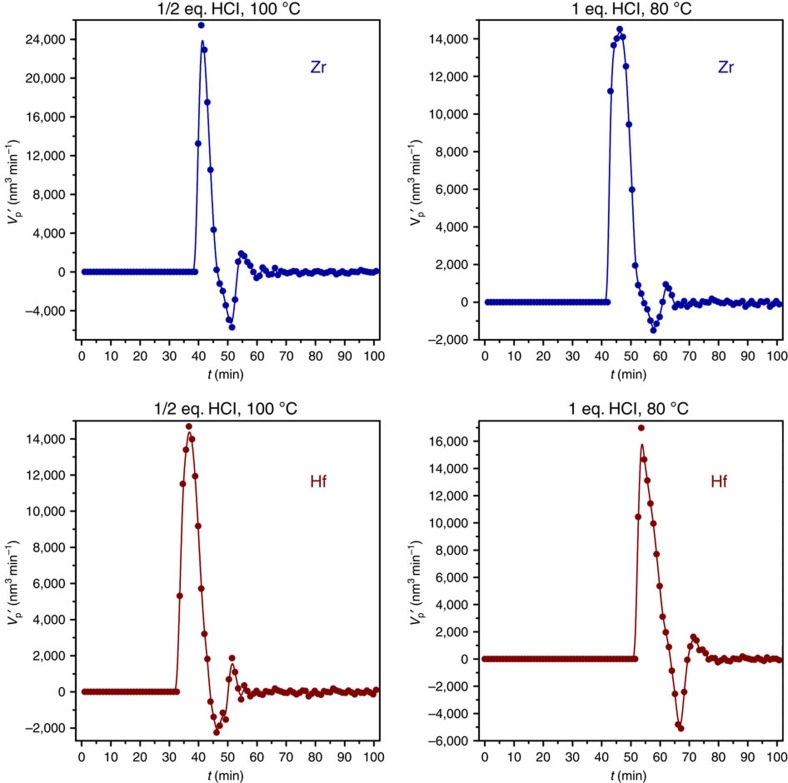
Particle growth rates. Time-resolved plot of particle growth rate, as monitored by *V*_p_*′* (nm^3^ min^−1^) for zirconium (blue) and hafnium (red) based UiO-66, at the conditions of ½ eq. HCl and 100 °C, and 1 eq. HCl and 80 °C.

Figure 1 displays the time derivative of the calculated Porod volume, *V*_p_*′* (or d*V*_p_*/*d*t*), versus time. *V*_p_*′* is used as a parameter for the particle growth rate. With Zr in blue and Hf in red, the evolution is displayed for the slower syntheses; ½ eq. HCl at 100 °C and 1 eq. HCl at 80 °C (eq. concerns a defined equivalent of HCl specified in the Methods section). We see that after a certain induction time and rapid condensation thereafter, the particle size oscillates to final values, that is, the derivative in Fig. 1 oscillates to zero. Oscillations were, in fact, found for the trends of all integral parameters (Supplementary Figs 10–14), and fully reproducible over several numbers of experiments. There is a difference between Zr and Hf in terms of dependence of maximum growth rate on temperature and HCl concentration. For Zr, decreasing the temperature by 20 °C and doubling the amount of HCl leads to a decrease of this maximum, whereas for Hf, there is only a small change and it follows the opposite direction. In further analysing the patterns, we found the evolution of the Porod volume to mimic the response of an underdamped harmonic oscillator. All trends, with exception of the fastest syntheses at 120 °C or 2 eq. of HCl, could be well fitted to *V*(*t*)=exp[−*γt*]·*a*·cos(*ωt*−*α*), where *γ* (min^−1^) is the damping coefficient, *ω* (min^−1^) the frequency and *a* (−) and *α* (min^−1^) amplitude and offset constants, respectively (see Supplementary Methods and Supplementary Fig. 1 for a more detailed explanation). What follows directly from our mathematical analysis is that syntheses involving higher temperatures and/or higher concentrations of HCl have their oscillations more effectively damped, and induction times are decreased. This is expected for a chemical clock: increasing the concentration of the autocatalytic species, generally, carries the same effect as increasing the temperature: the rate of the overall reaction goes up. It is interesting to check the response of oscillation frequency *ω* to changes in temperature and HCl concentration for both metals, by comparing it to the natural frequency *ω*_0_, which is the frequency of the undamped system. In doing so, Supplementary Fig. 9, we see that Zr-based UiO-66 reacts more strongly to an increase of temperature and HCl concentration with respect to Hf, and its oscillations are more easily damped. So, there are differences between Zr and Hf. This, and certainly Hf's sluggish behaviour with respect to Zr, is not new in coordination chemistry and has been observed before^20^.

Let us go back to the oscillations of all integral parameters; we remark that the final, equilibrium values for particle dimensions (*V*_p_, *R*_g_, *S*/*V*) in synthesis were found to be independent of temperature and or HCl concentration, consistent with scanning electron microscope analysis on the UiO-66 crystals by Farha and colleagues^19^. Notably, no oscillatory behaviour was observed for the extent of crystallization *α* obtained from the WAXS data, which is in line with observations by Ragon *et al*.^21^ in a study on formation of UiO-66 with *in situ* synchrotron X-ray diffraction. Moreover, the start of bulk crystallization was in all cases seen to carry a small delay, while proceeding gradually with respect to the process of condensation. This indicates that the condensed coordination polymer is briefly amorphous before rearranging towards a crystalline structure.

It is now important to explain the induction time and ensuing oscillatory behaviour, which is something expected for clock reactions, such as the clock reaction discussed in the introduction. In that reaction, iodide served as autocatalytic species serving an overall reaction without being consumed. The only species in the synthesis of UiO-66 that could carry such a role is HCl. A starting point in explaining the influence of HCl lies in the observation that as**-synthesized UiO-66 contains four μ_3_-OH ligands, next to four μ_3_-O ligands. This is striking, because the μ_3_-OH ligand is known to be highly Brønsted acidic. Indeed, in the case of UiO-66, it has been used to play either an active role in acid catalysis^22^, or be prone to exchange for other monovalent moieties in targeted functionalization of the inorganic node^23^. These ligands are thus expected to materialize in rather acidic local environments, which implies that HCl might be actively protonating the inorganic cluster during the synthesis of UiO-66. This by itself does not explain the clock-like phenomenon, for which HCl needs to be released by the crystallizing solid during the oscillations. But since we know that only four out of eight bridging oxygen ligands are protonated in the final framework for charge-balancing reasons, this would imply that in the short period during which the solid is amorphous, the number of protonated oxygen ligands could exceed the number of four. Thus, the question is whether protonated inorganic clusters may be vital during UiO-66 synthesis. In fact, this would not be a major surprise, for the decoration of building blocks by small moieties to promote MOF topologies is commonplace. For instance, we have shown by quantum chemical calculations before that the addition of an amine group to terephthalate is important for the ligand's ability to coordinate to the μ_3_-O-centred Al_3_ tectonic unit that builds NH_2_-Material Institute Lavoisier (MIL)-101(Al)^15^. Despite confirmation of experiment by computation, this, and analogous cases, have remained unexplained from the perspective of chemical theory, for calculations have merely focused on quantitative determination of energies instead of qualitative understanding^14^.

As Kohn–Sham density functional theory (DFT) is a molecular orbital (MO) method, it is insightful to picture the metal–organic bond as an MO interaction between an inorganic and an organic fragment, the nature of which we will try to unravel physically. We introduce the metal–organic MO (MOMO) analysis, which we believe will be of general use in providing chemically intuitive interpretation of DFT computation on MOF self-assembly. Herein, it is employed to investigate metal–organic interaction as function of degree of protonation of the inorganic unit.

### Density functional calculations

All calculations were carried out with Amsterdam density functional (ADF) at the ZORA-Becke-Lee-Yang-Parr (BLYP)-D3(BJ)/TZ2P level of the generalized gradient approximation (GGA) of Kohn–Sham DFT^24^,^25^,^26^, where ZORA stands for zeroth-order regular approximation, used to take relativistic effects into account, expected to be particularly important for calculations involving hafnium^27^,^28^. More details are given in the Methods section. In this first use of the MOMO method, a fragment of 12 geometrically optimized formate molecules is deformed to its final, crystallographic state in the UiO-66 framework using the activation-strain model^29^. The final crystallographic state is derived from Rietveld refinements provided by Lamberti *et al*.^30^,^31^ (Supplementary Table 3). Formate, rather than terephthalate or benzoate, was chosen to make the high-level calculations less expensive. The specified [formato]_12_ fragment is allowed to interact with an inorganic fragment of the form M_6_O_8_H_x_, with M=Zr or Hf, and *x*=0–8, rendering a total of 2 × 9 isoelectronic (inorganic) fragments, and 18 metal–organic interactions with the degree of protonation as main parameter in the construction of Zr- and Hf-based UiO-66. As explained above, it is our target to obtain a physical picture of the bonding situation.

**Figure 2:**
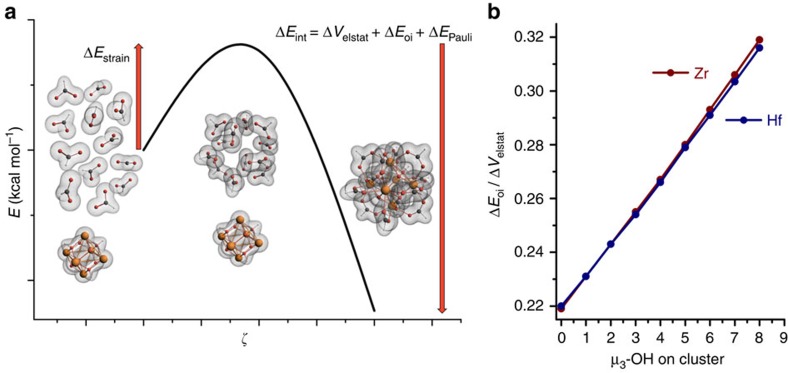
Activation strain model and degree of covalency. (**a**) Implementation of the activation-strain model and energy decomposition analysis (EDA), in which the [formato]_12_ fragment reacts with 18 isoelectronic M_6_O_8_H_x_ (*x*=0..8) fragments. ζ represents the reaction coordinate. The fragments deform towards their final state in the framework, and this metal–organic interaction is decomposed into energetic terms for electrostatic interaction, orbital interaction and Pauli repulsion. (**b**) Degree of covalency defined as Δ*E*_oi_*/*Δ*V*_elstat_ as calculated for the 18 fragment interactions (nine for zirconium and nine for hafnium based UiO-66).

We used an energy decomposition analysis (EDA) to analyse the metal–organic interaction. This allows us to split the energy associated with interaction into electrostatic, orbital and Pauli terms: Δ*E*_int_*=*Δ*V*_elstat_*+*Δ*E*_oi_*+*Δ*E*_Pauli_ (Fig. 2a)^32^.

As expected, the bonding becomes stronger upon protonation of the inorganic fragment. This is anticipated, because it involves increase of charge difference between the fragments. But it is here that EDA manifests itself as a valuable tool by providing the possibility of calculating a measure of covalency, herein defined as Δ*E*_oi_/Δ*V*_elstat_. This term is seen to increase linearly and significantly upon increasing the degree of protonation of the inorganic fragment (Fig. 2b). Clearly, highly protonated clusters engage in stronger orbital interactions and precipitate more irreversibly. We relate this to the additional strengthening of the bond, and arguably also the reduction in polarity that prevents solvent attack on the bond, and thus also dissociation and solvation. Mulliken populational analysis (Supplementary Table 2) also indicates that electron transfer from the [formato]_12_ fragment increases upon increasing the number of protons on the (electron-accepting) inorganic fragment.

**Figure 3:**
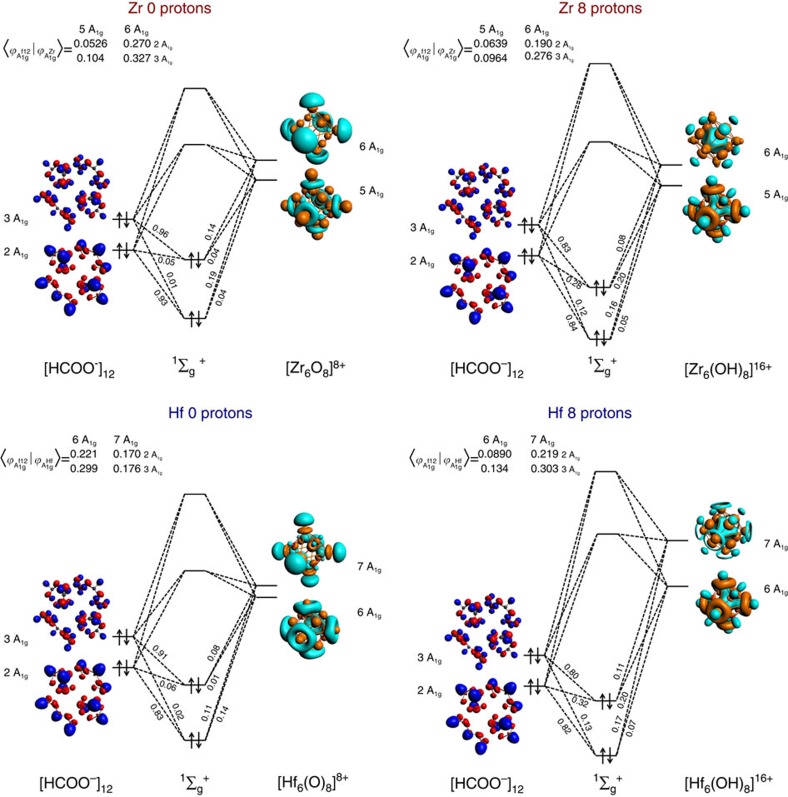
MOMO diagrams. MOMO diagrams showing the metal–organic interaction between the totally symmetric (A_1g_) fragment orbitals, with the inorganic fragment either carrying zero or eight protons, in *O*_*h*_ symmetry. The overlap matrices are depicted in the top left corner for each diagram.

The MOMO approach provides insight in disclosing the nature of this increase in metal–organic donor–acceptor interaction. We first recognize that the bonding between the two fragments involves 36 electron pairs (24*σ* and 12*π*, in line of expectation for 12 μ_2_-formato ligands). We then note that in many cases the orbital interactions between the two fragments involve more than two fragment orbitals, which makes it rather challenging to construct a simple MO picture that provides a good qualitative picture of the influence of protonation on the metal–organic bond formation. However, if one only considers the interactions involving the inorganic fragments with zero and eight protons, one does not only consider the extremes that span the range of study, but also the interactions in which both interacting fragments carry *O*_*h*_ symmetry. This considerably facilitates the analysis: the use of group theory indicates that solely considering the high symmetries A_1g_, A_2g_, A_2u_, thus three MO diagrams per model complex, a reasonably complete picture can be obtained. Only the four MOs of totally symmetric A_1g_ are displayed in Fig. 3, since they represent the most dominant interactions (MO diagrams for A_2g_ and A_2u_ can be found in Supplementary Figs 15–23 and Supplementary Table 1). From the MO coefficients displayed in Fig. 3, it is clear that for both Zr- and Hf-based UiO-66, metal–organic bonding becomes decreasingly polar as the degree of protonation increases. Note that the stronger bonding is not a result of the metal–organic orbital overlap, which remains similar if not slightly decreasing for the eight proton inorganic clusters with respect to their zero proton analogues.

The analysis of the metal–organic bonding thus indicates that while bonds are spatially similar, covalency—as we measured it—increases. In a simple description of this phenomenon, we consider the postulate of the bond-order conservation principle, without the Morse-potential approach as frequently applied by Shustorovich and others^33^, and observe the inorganic cluster that undergoes protonation at μ_3_-O. According to Shustorovich' theory this weakens the metal–oxygen bond and therefore increases the acidity of the metal ion, thus enhancing the metal–organic interaction in this case.

## Discussion

**Figure 4:**
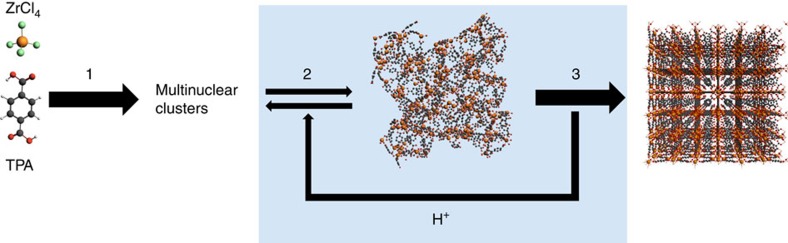
Schematic view of the formation of UiO-66 and the autocatalytic role of H^+^. Step 1: formation of multinuclear clusters; step 2: consumption of H^+^ and fast condensation; and step 3: development of crystallinity upon release of H^+^.

Figure 4 displays a schematic pathway based on the observations in SAXS/WAXS and DFT analysis. Step 1 in the scheme occurs in the preparation of the precursor solution and concerns the water-promoted hydrolysis of ZrCl_4_ and formation of zirconyl chloride species, which are known to form multinuclear species in solution, such as [Zr_4_(μ_2_-OH)_8_]^8+^^34^. The rearrangement of such clusters into the hexanuclear UiO-66 building block follows an unknown path, but what is certain is that the pathway to condensation must be both water and acid catalysed, since in the appearance of μ_3_-O(H) bridges, O's remain largely protonated. This is indeed observed by the groups of Serre and Farha^19^,^21^. The highly protonated clusters then engage in metal–organic bond formation in step 2 and fast condensation of the solid coordination polymer ensues. The solid is not fully crystalline at this brief stage, and the situation is metastable. Reorganization however, evidenced by SAXS versus WAXS comparison, is swift: the crystalline UiO-66 lattice is formed and the oxido bridges deprotonate, leaving the scaffold charge-neutralized (step 3). This process of bulk crystallization within the solid state deserves some notion. By virtue of established laws in solid-state chemistry, such rapid transition requires a large degree of chemical inheritance between parent and resulting solid, that is, involving no substantial bond breakage and reformation^35^. Thus, in line with works by Cheetham^36^, we expect the solid to exist as amorphous MOF, chemically cognate to UiO-66, but with protonated oxido bridges countered by chloride ions in solution.

The drive behind the process of crystallization is in all likelihood caused by lattice enthalpy, similar to what we observed before in the crystal-to-crystal transformation of NH_2_-MOF-235(Al) to NH_2_-MIL-101(Al)^15^, and also seen in the formation of zeolites, where charged templates are squeezed out of the network during the process of crystallization^37^. The release of protons in step 3 is vital, since it accelerates step 2, which is prohibited from moving forward, and even reversed by the low concentration of protons after their consumption. This is witnessed by the negative values for V_*p*_*′* in Fig. 1. The oscillating proton concentration, caused by the coupling of step 2 and step 3, thus leads to oscillating temporary equilibria in step 2, and dissolution recrystallization near the surface of the particle governs commensurate oscillations for all integral parameters.

Despite this being the first report on a clock reaction that generates oscillations in the condensed state, we do not rule out the possibility that similar processes occur in the formation of other MOFs; on the contrary, we expect them to be present in the coordination of other high-coordinate oxoclusters.

Again, this work demonstrates that in resolving molecular pathways of MOF crystallization, a multi-scale approach is required: in this work, the use of both SAXS and WAXS was required to separate the process of the condensation from crystallization and show them to exist as independent processes in MOF synthesis. At the molecular scale, the MOMO Kohn–Sham DFT approach extends the information obtained with X-ray scattering to provide necessary insight from the perspective of coordination chemistry. We believe that the MOMO approach should be encouraged in theoretical studies on MOF formation, since interaction between the organic struts and inorganic nodes can be highlighted, essential in the understanding of metal–organic network formation. It is herein demonstrated that this is a viable new theoretical approach, while overall, the result adds to the exciting array of phenomena associated with nonlinear dynamics.

## Methods

### Synthesis

An in-house developed SAXS/WAXS cell (Supplementary Figs 2 and 3), further described elsewhere^16^, was used for the experiments. For a typical solution, 0.75 mmol terephthalic acid (134 mg), 0.54 mmol anhydrous ZrCl_4_/HfCl_4_ (125/172 mg), 1 ml HCl (37%) and 15 ml dried dimethylformamide (DMF) were mixed and injected in the synchrotron cell. A measure of 1 ml stands for 1 eq. of HCl in the text, added to the starting solution.

### SAXS/WAXS measurements

Time-resolved SAXS/WAXS experiments were performed at the X9 beamline at the NSLS at Brookhaven National Laboratory (New York, USA). The undulator-based X9 beamline at the NSLS applied a marCCD SAXS detector and photonic science WAXS detector. The wavelength was in all cases 0.879 Å, and the sample-to-detector distance was 3.099 and 0.4500, m for the SAXS and WAXS camera, respectively. In the cell, the synthesis solution is loaded between two mica windows separated by polytetrafluoroethylene (PTEF) spacers (1.5 mm thickness). The depth of the solution probed by X-rays in measurement was ca. 75 mm. The beam-spot size was 20 by 20 μm. Heating was provided by four cartridge electrical resistances and temperature was controlled at the external wall of the PTFE inserts with a temperature controller.

In calculation of the integral parameters, the Guinier regime for the calculation of *R*_g_ was for all data sets based on the scattering vector range of *q*=(0.07–0.15), the Porod regime was in all cases *q*=(3.2…4). The number density is based on both the Guinier and Porod regime.

All data sets were background subtracted, azimuthally integrated and normalized for beam intensity to yield time-resolved one-dimensional data that was processed to obtain temporal information on the following integral parameters: radius of gyration *R*_g_ (nm),


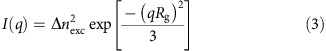


Porod volume *V*_p_ (nm^3^)


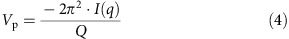


with Porod invariant *Q*


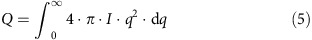


Porod surface-to-volume *S/V* (nm^−1^)


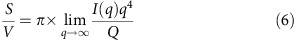


and number densiy *N/V* (−)


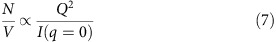


Where forward scattering*, I*(*q=0*) was calculated using (3) to extrapolate *q* to 0.

Extent of crystallization *α* was derived from the most intense Bragg peak in UiO-66, at *q*=0.5 nm^−1^. Supplementary Figures 3–8 show the *in situ* collected diffraction data.

### DFT calculations

All standard calculations were performed using the ADF program^27^,^28^,^37^,^38^, using the BLYP functional. The numerical integration was performed using the procedure developed by Boerrigter, te Velde and Baerends^39^,^40^. The MOs were expanded in a large uncontracted set of Slater type orbitals containing diffuse functions, which is of triple-*ζ* quality for all atoms and was augmented with two sets of polarization functions (TZ2P). The core shells of all atoms were treated by the frozen-core approximation^41^. All energies and geometries were calculated using the GGA of DFT at the BLYP level^42^,^43^. GGA proceeds from the local density approximation, where exchange is described by Slater's X*α* potential and correlation is treated in the Vosko–Wilk–Nusair parametrization^44^, which is augmented with nonlocal corrections to exchange due to Becke^45^, and correlation due to Perdew^46^ added self-consistently^47^. For all calculations, relativistic effects were accounted for by ZORA^48^ and dispersion by Grimme's correction, D3^45^. The (small) contribution from dispersion, obtained via EDA can be found in the Supplementary Information.

The MOMO approach is based on the activation-strain model, elaborated on elsewhere^32^,^49^,^50^,^51^. Activation-strain model was only applied to the organic fragment, since the unprotonated inorganic fragment is not stable upon geometry optimization. To verify that formate ligands are representative in calculation, an additional (expensive) calculation with four protons and benzoate fragments was carried out at the same computational level.

### Data availability

The authors declare that all other relevant data not included in the Supplementary Information and supporting the findings of this study are available on request.

## Additional information

**How to cite this article:** Goesten, M. G. *et al*. Evidence for a chemical clock in oscillatory formation of UiO-66. *Nat. Commun.* 7:11832 doi: 10.1038/ncomms11832 (2016).

## Supplementary Material

Supplementary Information Supplementary Figures 1-23, Supplementary Tables 1-3, Supplementary Methods and Supplementary Reference

## References

[b1] Lente G., Bazsa G. & Fábián I. What is and what isn't a clock reaction? New J. Chem. 31, 1707 (2007).

[b2] Epstein I. & Pojman J. An Introduction to Nonlinear Chemical Dynamics : Oscillations, Waves, Patterns, and Chaos: Oscillations, Waves, Patterns, and Chaos Oxford Univ. Press (1998).

[b3] *LearnChemistry, Iodine clock reaction.* (Nuffield Foundation and the Royal Society of Chemistry, 2015) Available at http://www.rsc.org/learn-chemistry/resource/res00000744/iodine-clock-reaction?cmpid=CMP00005152.

[b4] Orbán M., Kurin-Csörgei K. & Epstein I. R. pH-regulated chemical oscillators. Acc. Chem. Res. 48, 593–601 (2015). 25705810 10.1021/ar5004237

[b5] Yoshida R., Takahashi T., Yamaguchi T. & Ichijo H. Self-Oscillating Gel. J. Am. Chem. Soc. 118, 5134–5135 (1996).

[b6] Howse J. R. . Reciprocating power generation in a chemically driven synthetic muscle. Nano Lett. 6, 73–77 (2006). 16402790 10.1021/nl0520617

[b7] Epstein I. R. & Pojman J. A. Overview: nonlinear dynamics related to polymeric systems. Chaos 9, 255–259 (1999). 12779822 10.1063/1.166401

[b8] Billingham J. & Coveney P. V. Simple chemical clock reactions: application to cement hydration. Faraday Trans. 89, 3021 (1993).

[b9] Surblé S., Millange F., Serre C., Férey G. & Walton R. I. An EXAFS study of the formation of a nanoporous metal-organic framework: evidence for the retention of secondary building units during synthesis. Chem. Commun. 14, 1518–1520 (2006). 10.1039/b600709k 16575446

[b10] Cravillon J. . Fast nucleation and growth of ZIF-8 nanocrystals monitored by time-resolved *in situ* small-angle and wide-angle X-ray scattering. Angew. Chem. Int. Ed. 50, 8067–8071 (2011). 10.1002/anie.201102071 21748830

[b11] Millange F., Osta, El R., Medina M. E. & Walton R. I. A time-resolved diffraction study of a window of stability in the synthesis of a copper carboxylate metal-organic framework. CrystEngComm 13, 103–108 (2011).

[b12] Millange F. . Effect of the nature of the metal on the breathing steps in MOFs with dynamic frameworks. Chem. Commun. 4732–4734 (2008). 10.1039/b809419e 18830475

[b13] Millange F. . Time-resolved *in situ* diffraction study of the solvothermal crystallization of some prototypical metal-organic frameworks. Angew. Chem. Int. Ed. 49, 763–766 (2010). 10.1002/anie.200905627 20017176

[b14] Goesten M. G., Kapteijn F. & Gascon J. Fascinating chemistry or frustrating unpredictability: observations in crystal engineering of metal-organic frameworks. CrystEngComm 15, 9249–9257 (2013).

[b15] Goesten M. G. . Molecular promoting of aluminum metal-organic framework topology MIL-101 by N,N-dimethylformamide. Inorg. Chem. 53, 882–887 (2014). 24405155 10.1021/ic402198a PMC4051174

[b16] Stavitski E. . Kinetic control of metal-organic framework crystallization investigated by time-resolved *in situ* X-ray scattering. Angew. Chem. Int. Ed. 50, 9624–9628 (2011). 10.1002/anie.201101757 21761517

[b17] Yaghi O. M. . Reticular synthesis and the design of new materials. Nature 423, 705–714 (2003). 12802325 10.1038/nature01650

[b18] Férey G. Hybrid porous solids: past, present, future. Chem. Soc. Rev. 37, 191–214 (2008). 18197340 10.1039/b618320b

[b19] Katz M. J. . A facile synthesis of UiO-66, UiO-67 and their derivatives. Chem. Commun. 49, 9449 (2013). 10.1039/c3cc46105j 24008272

[b20] Marschner C. Hafnium: stepping into the limelight!. Angew. Chem. Int. Ed. 46, 6770–6771 (2007). 10.1002/anie.200702573 17712867

[b21] Ragon F. . *In situ* energy-dispersive x-ray diffraction for the synthesis optimization and scale-up of the porous zirconium terephthalate UiO-66. Inorg. Chem. 53, 2491–2500 (2014). 24527942 10.1021/ic402514n

[b22] Vandichel M. . Active site engineering in UiO-66 type Metal Organic Frameworks by intentional creation of defects : a theoretical rationalization. CrystEngComm 17, 395–406 (2015).

[b23] Larabi C. & Quadrelli E. A. Titration of Zr 3(μ-OH) hydroxy groups at the cornerstones of bulk MOF UiO-67, [Zr 6O 4(OH) 4(biphenyldicarboxylate) 6], and their reaction with [AuMe(PMe 3)]. Eur. J. Inorg. Chem. 3014–3022 (2012).

[b24] Velde te G. . Chemistry with ADF. J. Comput. Chem. 22, 931–967 (2001).

[b25] Computer code ADF, . Baerends E. J., . Scientific Computing and Modelling Vrije Universiteit Amsterdam (2014).

[b26] Fonseca Guerra C., Handgraaf J.-W., Baerends E. J. & Bickelhaupt F. M. Voronoi deformation density (VDD) charges: assessment of the Mulliken, Bader, Hirshfeld, Weinhold, and VDD methods for charge analysis. J. Comput. Chem. 25, 189–210 (2004). 14648618 10.1002/jcc.10351

[b27] Van Lenthe E., Baerends E. J. & Snijders J. G. Relativistic total energy using regular approximations. J. Chem. Phys. 101, 9783–9792 (1994).

[b28] Van Lenthe E., Van Leeuwen R., Baerends E. J. & Snijders J. G. Relativistic regular two-component hamiltonians. Int. J. Quantum Chem. 57, 281–293 (1996).

[b29] Fernández I. & Bickelhaupt F. M. The activation strain model and molecular orbital theory: understanding and designing chemical reactions. Chem. Soc. Rev. 43, 4953–4967 (2014). 24699791 10.1039/c4cs00055b

[b30] Valenzano L. . Disclosing the complex structure of UiO-66 metal organic framework: a synergic combination of experiment and theory. Chem. Mater. 23, 1700–1718 (2011).

[b31] Jakobsen S. . Structural determination of a highly stable metal-organic framework with possible application to interim radioactive waste scavenging: Hf-UiO-66. Phys. Rev. B 86, 125429 (2012).

[b32] Radius U., Bickelhaupt F. M., Ehlers A. W., Goldberg N. & Hoffmann R. Is CO a Special ligand in organometallic chemistry? Theoretical investigation of AB, Fe(CO) 4AB, and Fe(AB) 5(AB=N 2, CO, BF, SiO). Inorg. Chem. 37, 1080–1090 (1998).

[b33] Shustorovich E. Coverage effects under atomic chemisorption: Morse-potential modeling based on bond-order conservation. Surface Science 163, L645–L654 (1985).

[b34] Nielsen R. in Ullmann's Encyclopedia of Industrial Chemistry Wiley-VCH Verlag GmbH & Co. KGaA (2000).

[b35] Baronnet A. Silicate microstructures at the sub-atomic scale. Comptes rendus de l'Academie des sciences. Serie II. Sciences de la terre et des planetes 324, 157–172 (1997).

[b36] Bennett T. D. & Cheetham A. K. Amorphous metal-organic frameworks. Acc. Chem. Res. 47, 1555–1562 (2014). 24707980 10.1021/ar5000314

[b37] Szyja B., Jansen A., Verstraelen T. & Van Santen R. Molecular dynamics study of the silica-water-SDA interactions. Phys. Chem. Chem. Phys. 11, 7605–7610 (2009). 19950499 10.1039/b822859k

[b38] Snijders J. G., Vernooijs P. & Baerends E. J. Roothaan-Hartree-Fock-Slater atomic wave functions: single-zeta, double-zeta, and extended Slater-type basis sets for 87Fr-103Lr. At. Data Nucl. Data Tables 26, 483–509 (1981).

[b39] Fonseca Guerra C., Snijders J. G., Velde, te G. & Baerends E. J. Towards an order-N DFT method. Theor. Chem. Acc. 99, 391–403 (1998).

[b40] Boerrigter P. M., Velde, te G. & Baerends J. E. Three-dimensional numerical integration for electronic structure calculations. Int. J. Quantum Chem. 33, 87–113 (1988).

[b41] Velde te, G. & Baerends E. J. Numerical integration for polyatomic systems. J. Comput. Phys. 99, 84–98 (1992).

[b42] Baerends E. J., Ellis D. E. & Ros P. Self-consistent molecular Hartree-Fock-Slater calculations I. the computational procedure. Chem. Phys. 2, 41–51 (1973).

[b43] Becke A. D. Density-functional exchange-energy approximation with correct asymptotic behavior. Phys. Rev. A 38, 3098–3100 (1988). 10.1103/physreva.38.3098 9900728

[b44] Lee C., Yang W. & Parr R. G. Development of the Colle-Salvetti correlation-energy formula into a functional of the electron density. Phys. Rev. B 37, 785–789 (1988). 10.1103/physrevb.37.785 9944570

[b45] Van Lenthe E., Snijders J. G. & Baerends E. J. The zero-order regular approximation for relativistic effects: The effect of spin-orbit coupling in closed shell molecules. J. Chem. Phys. 105, 6505–6516 (1996).

[b46] Vosko S. H., Wilk L. & Nusair M. Accurate spin-dependent electron liquid correlation energies for local spin density calculations: a critical analysis. Can. J. Phys. 58, 1200–1211 (1980).

[b47] Perdew J. Density-functional approximation for the correlation energy of the inhomogeneous electron gas. Phys. Rev. B 33, 8822–8824 (1986). 10.1103/physrevb.33.8822 9938299

[b48] Fan L. & Ziegler T. The influence of self-consistency on nonlocal density functional calculations. J. Chem. Phys. 94, 6057–6063 (1991).

[b49] Grimme S., Antony J., Ehrlich S. & Krieg H. A consistent and accurate ab initio parametrization of density functional dispersion correction (DFT-D) for the 94 elements H-Pu. J. Chem. Phys. 132, 154104-1–154104-18 (2010). 20423165 10.1063/1.3382344

[b50] Van Zeist W. J. & Bickelhaupt F. M. The activation strain model of chemical reactivity. Organ. Biomol. Chem. 8, 3118–3127 (2010). 10.1039/b926828f 20490400

[b51] Wolters L. P. & Bickelhaupt F. M. The activation strain model and molecular orbital theory. WIREs Comput. Mol. Sci. 5, 324–343 (2015). 10.1002/wcms.1221 PMC4696410 26753009

