# Supplementary material for: Evidence for a chemical clock in oscillatory formation of UiO-66
Source: Nat Commun. 2016 Jun 10;7:11832. doi: 10.1038/ncomms11832 (PMC4906383; doi:10.1038/ncomms11832)
Supplement: Supplementary Information — Supplementary Figures 1-23, Supplementary Tables 1-3, Supplementary Methods and Supplementary Reference [file ncomms11832-s1.pdf]

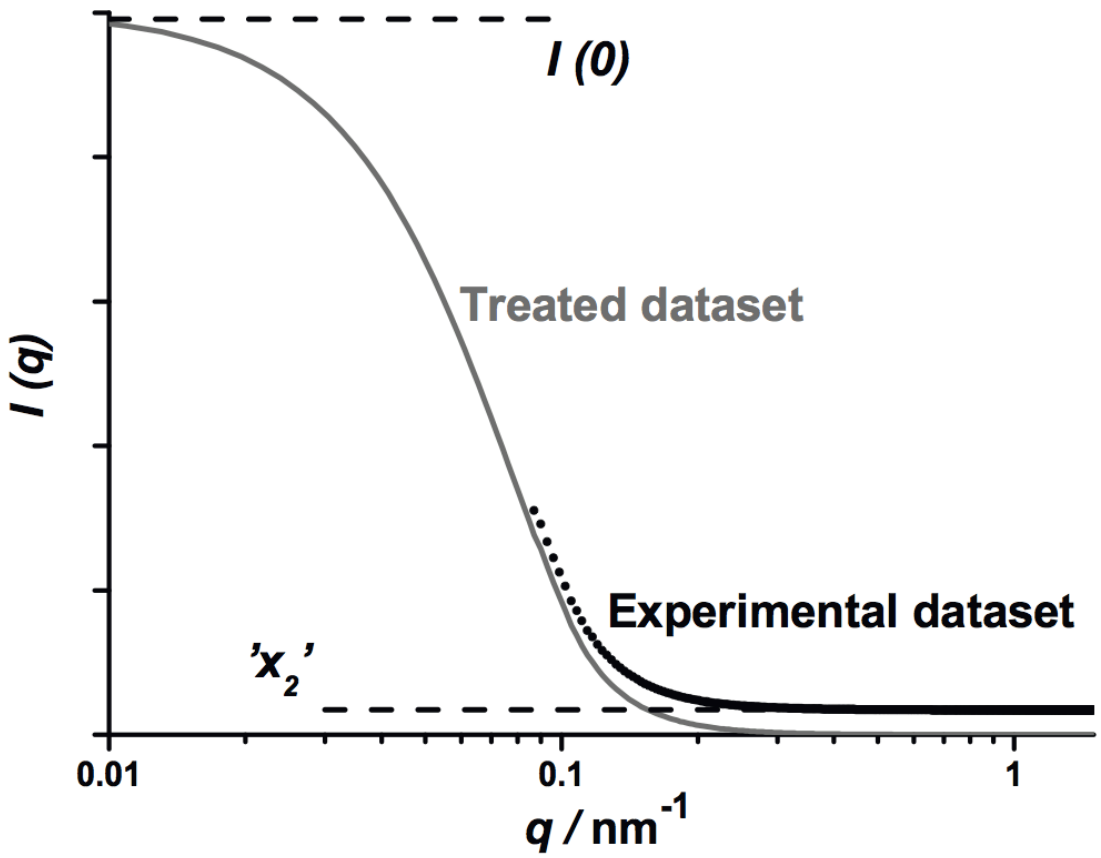

Supplementary Figure 1 Experimental and treated SAXS dataset of NH_2-MIL-101(Al), in an *I(q) vs. log(q)* plot. The treated dataset (gray) is the same as the experimental dataset, but has additional extrapolated values for *q* going to zero and for *q* going to infinity, background values are subtracted. Values for *I(0)* and background parameter x_2 are indicated.

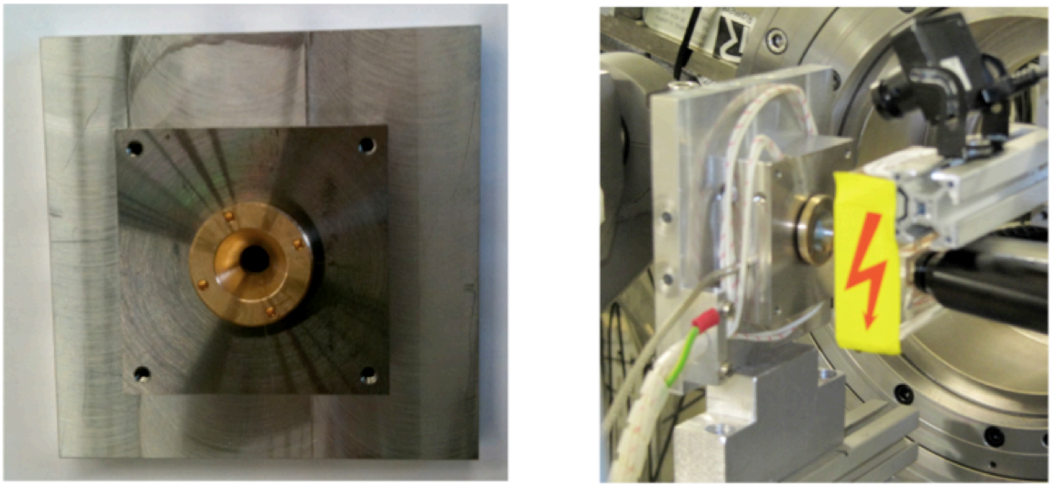

Supplementary Figure 2 *In-situ* X-ray Scattering measurement cell, left: backside, right: in operation

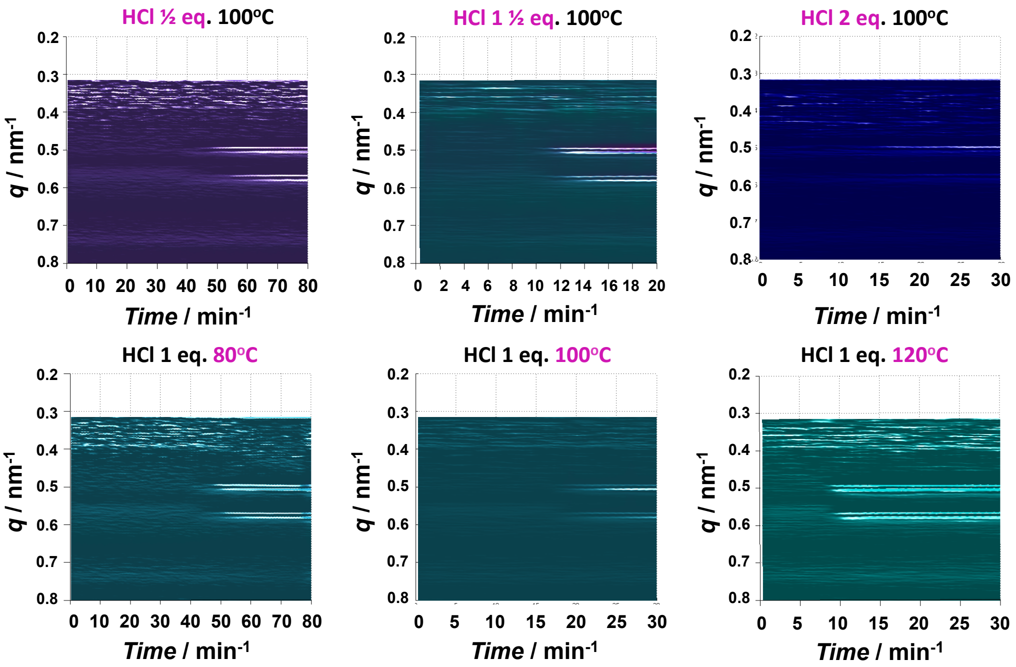

Supplementary Figure 3 In-situ WAXS data obtained for synthesis of UiO-66(Zr) in different solutions. “Eq.” represents equivalent (with 1 eq. representing the typical amount denoted above). The colours are arbitrary.

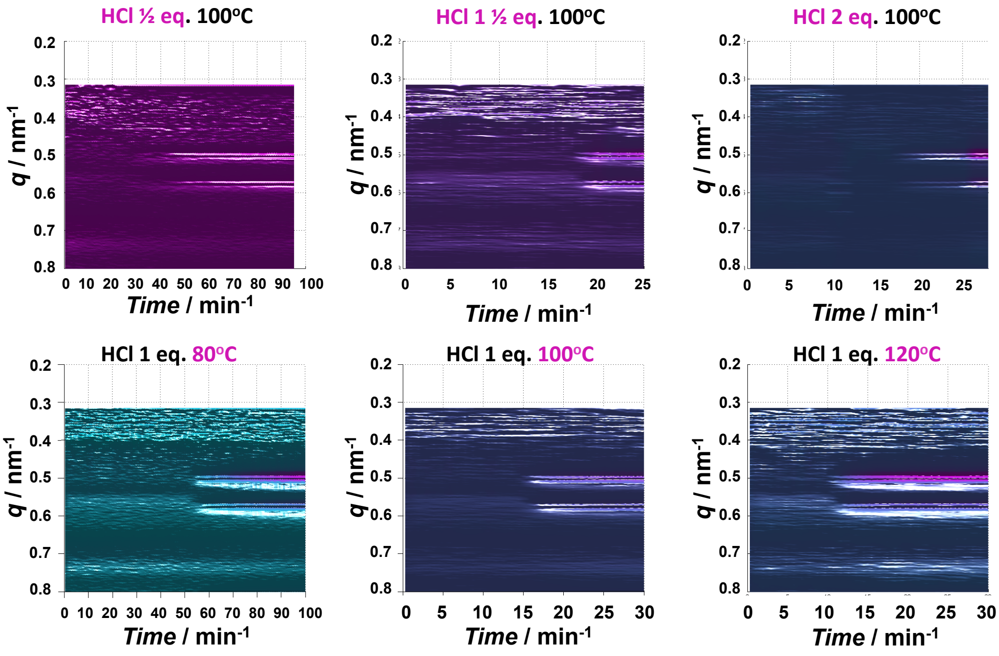

Supplementary Figure 4 In-situ WAXS data obtained for synthesis of UiO-66(Hf) in different circumstances.

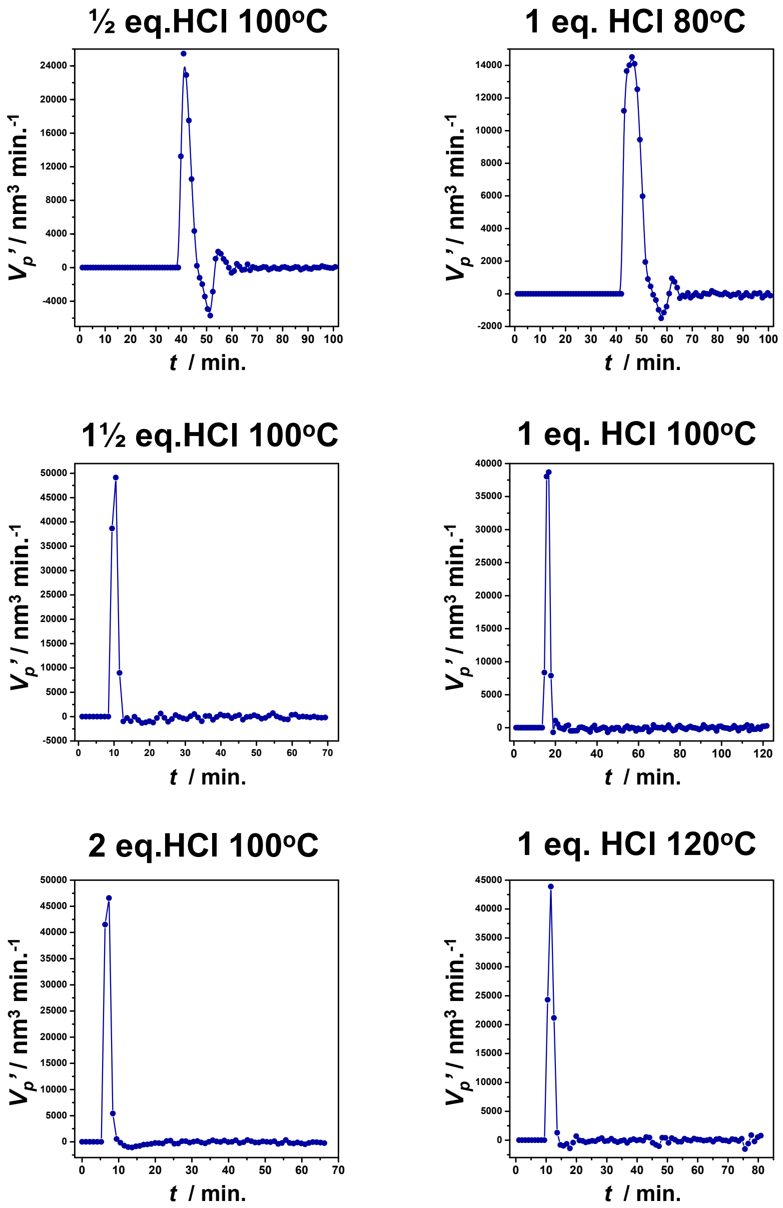

Supplementary Figure 5 dV/dt plots for the syntheses of Zirconium-based UiO-66.

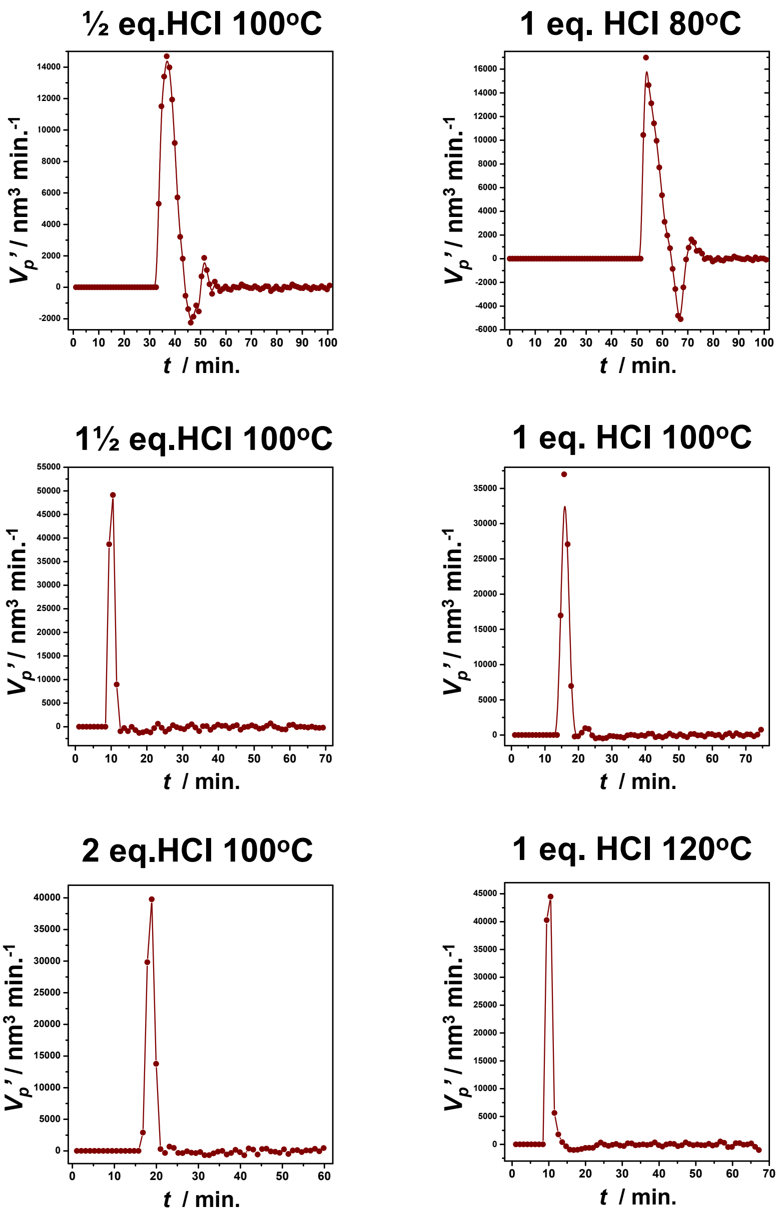

Supplementary Figure 6 dV/dt plots for the syntheses of Hafnium-based UiO-66.

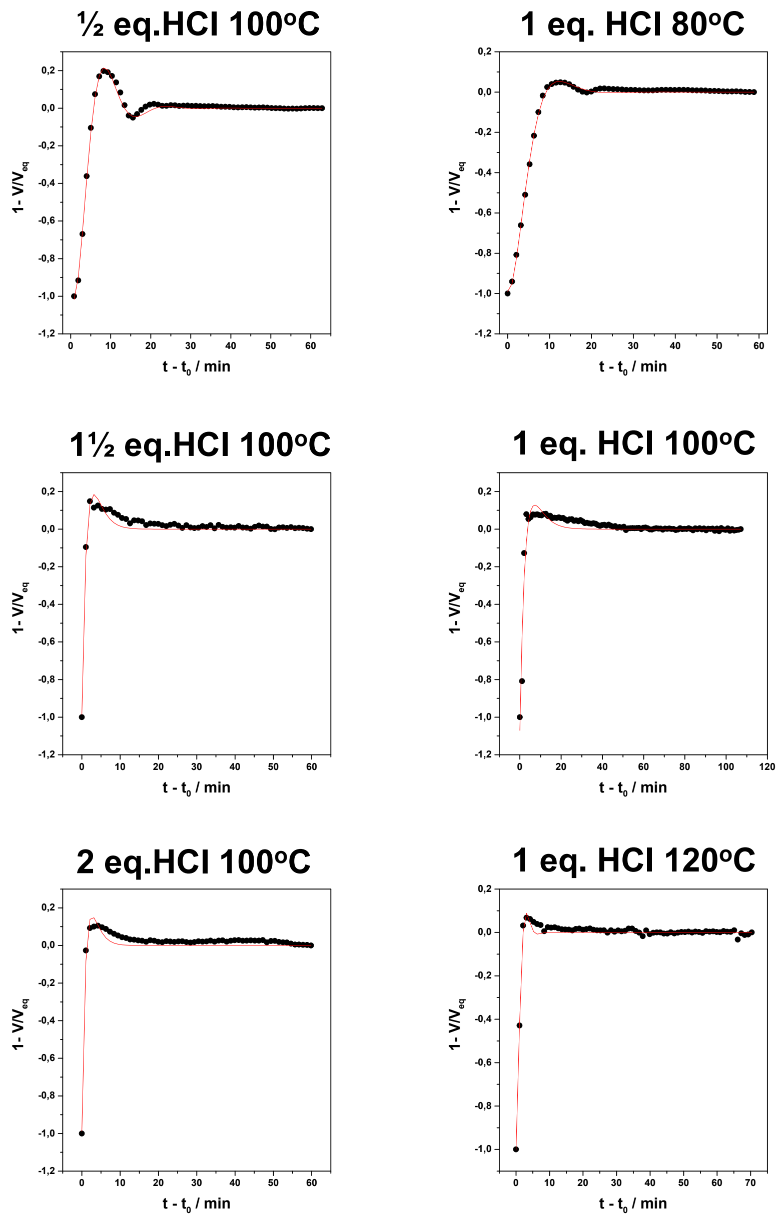

Supplementary Figure 7 With V_{eq} the equilibrium value of the Porod Volume, and t_0 the time value at which precipitation starts, fits obtained with the underdamped harmonic oscillator model for all Zirconium-based syntheses.

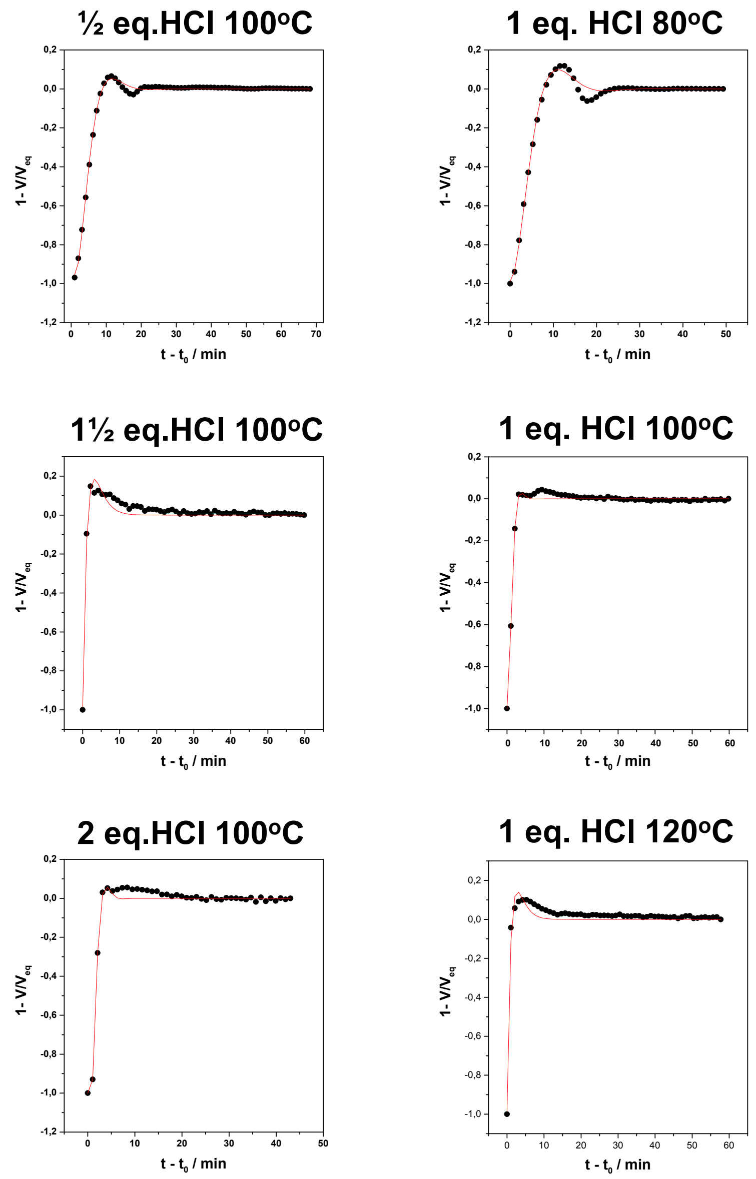

Supplementary Figure 8 Analogous to supplementary figure 7, fits obtained with the underdamped harmonic oscillator model for all Hafnium-based syntheses.

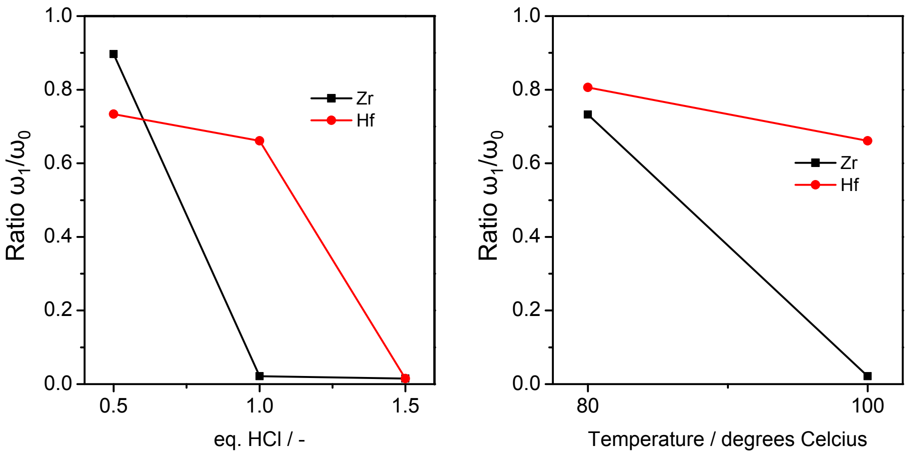

Supplementary Figure 9 ω/ω_0 response to changes in HCl and temperature.

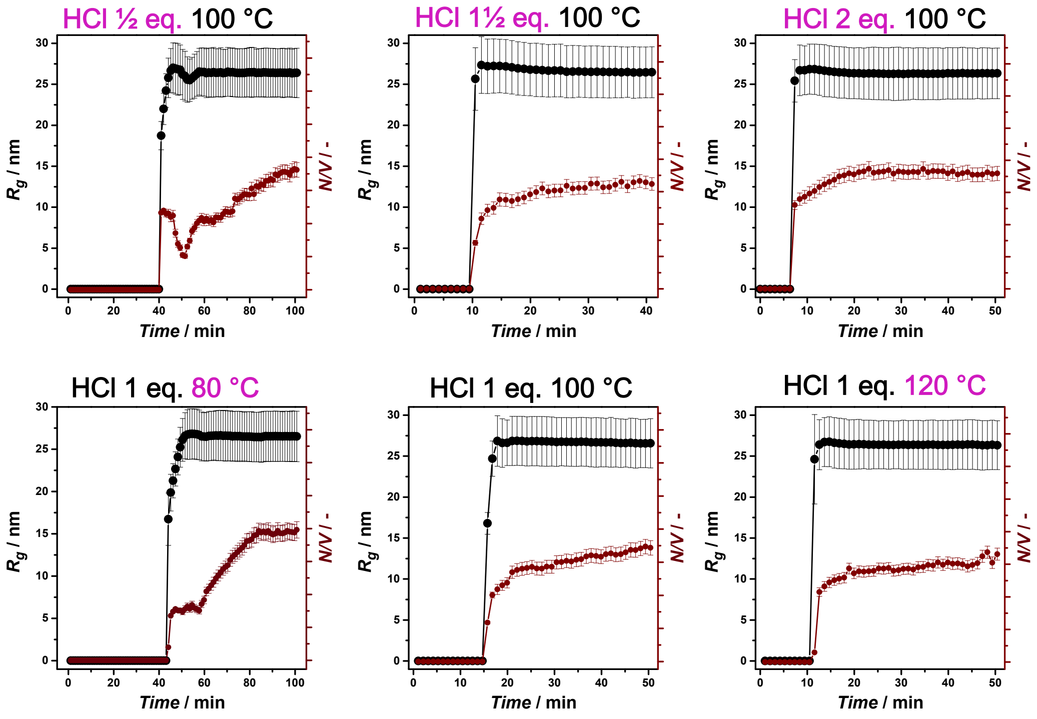

Supplementary Figure .10: temporal profiles for R_p and *N/V* calculated for the synthesis of Zirconium-based UiO-66.

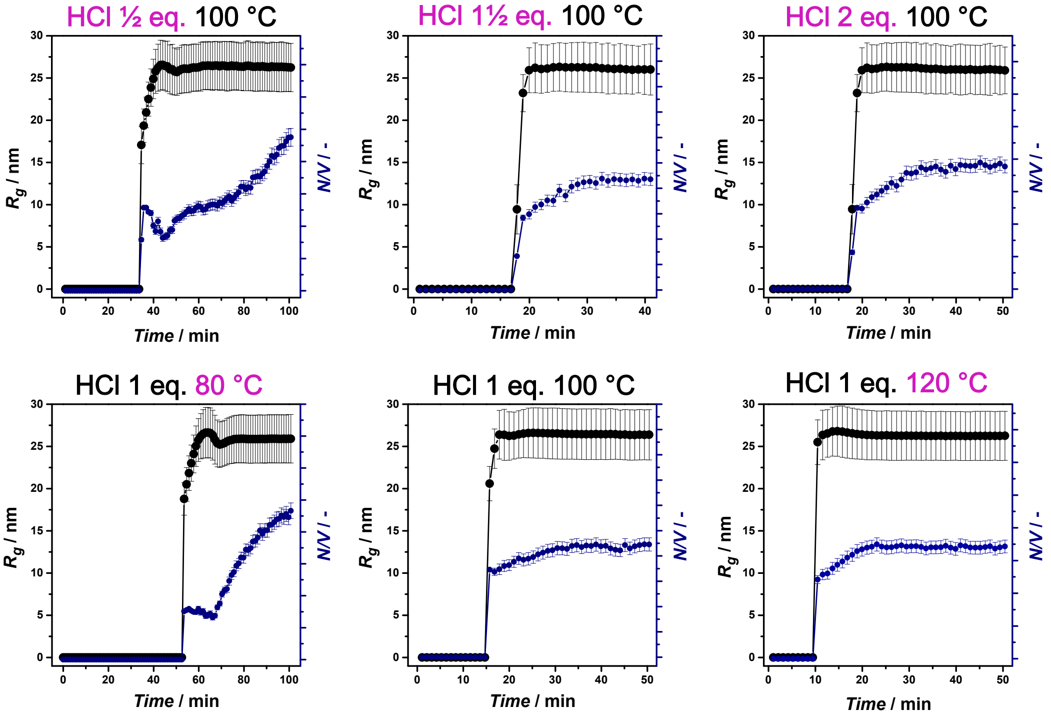

Supplementary Figure 11: Analogous to figure S.9, calculated for the synthesis of Hafnium-based UiO-66.

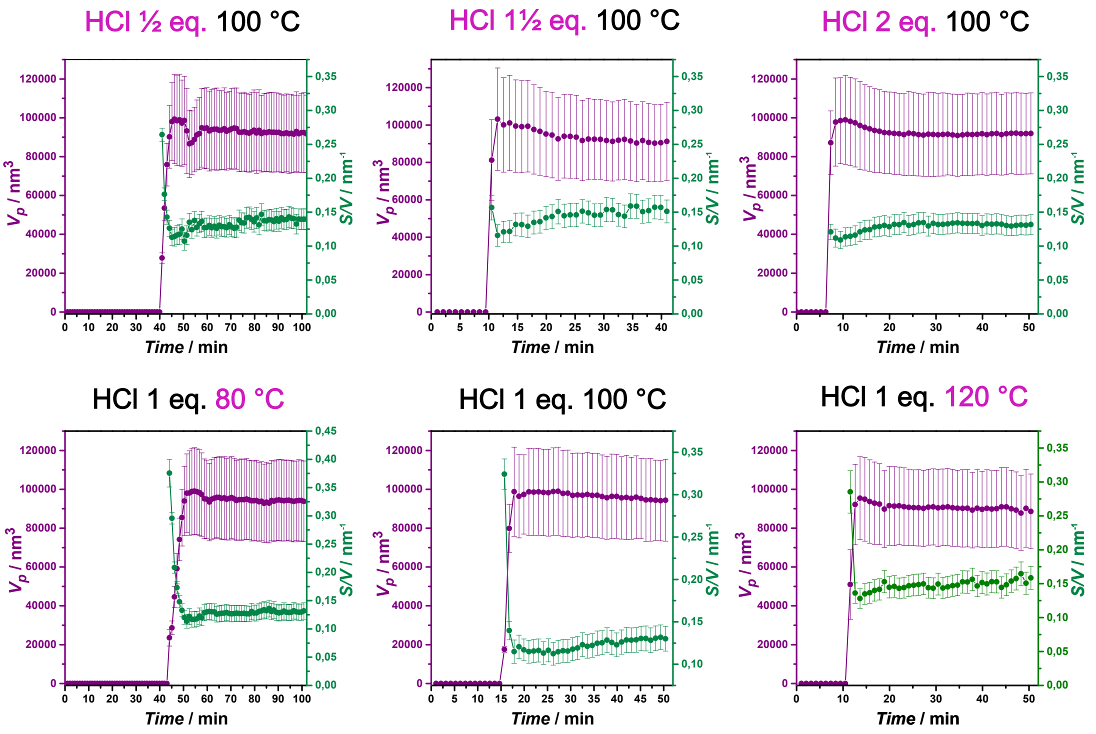

Supplementary Figure 12: temporal profiles for *S/V* and V_p calculated for the synthesis of Zirconium-based UiO-66.

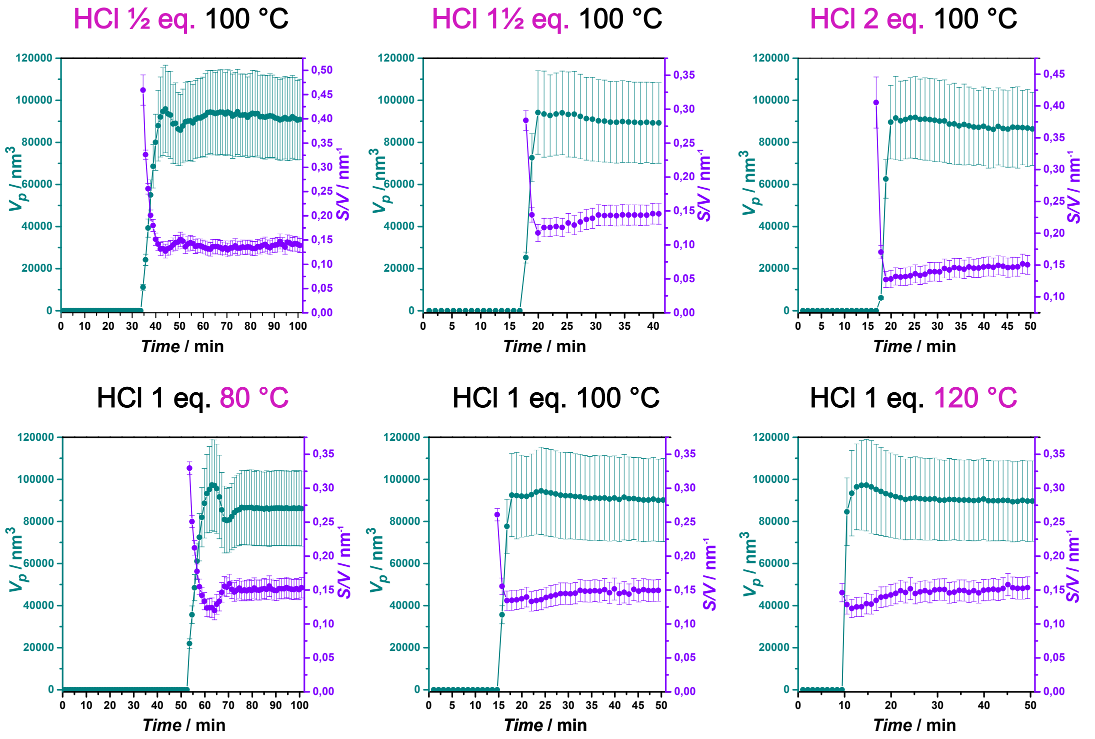

Supplementary Figure 13: Analogous to supplementary figure 11, calculated for the synthesis of Hafnium-based UiO-66.

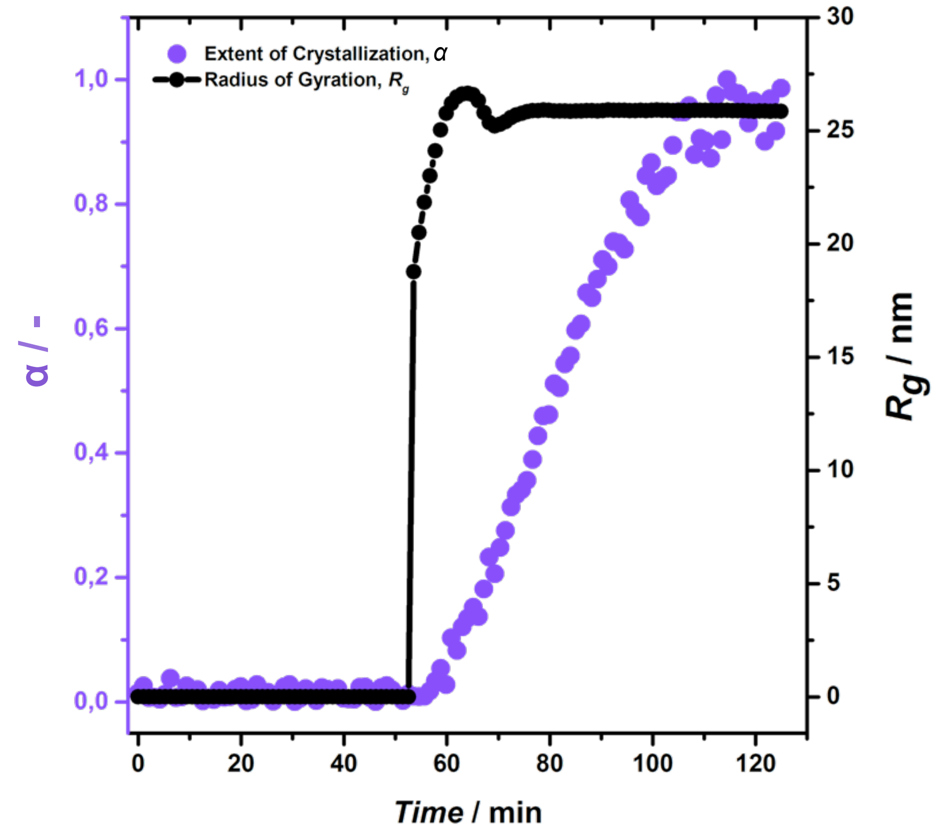

Supplementary Figure 14 Exemplary comparison between development in extent of crystallisation, α, and radius of gyration, R_g for the synthesis of UiO-66(Hf) at 80°C.

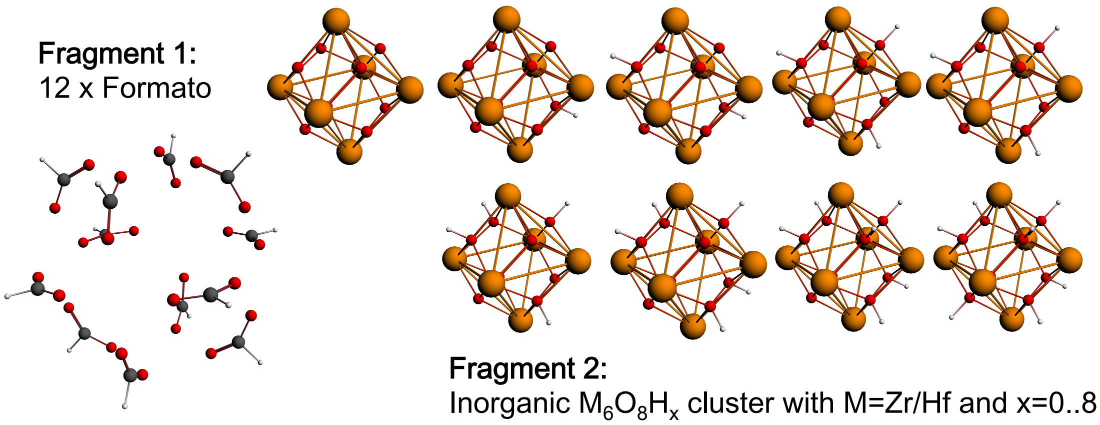

Supplementary Figure 15 Definition of the fragments discussed in the main text.

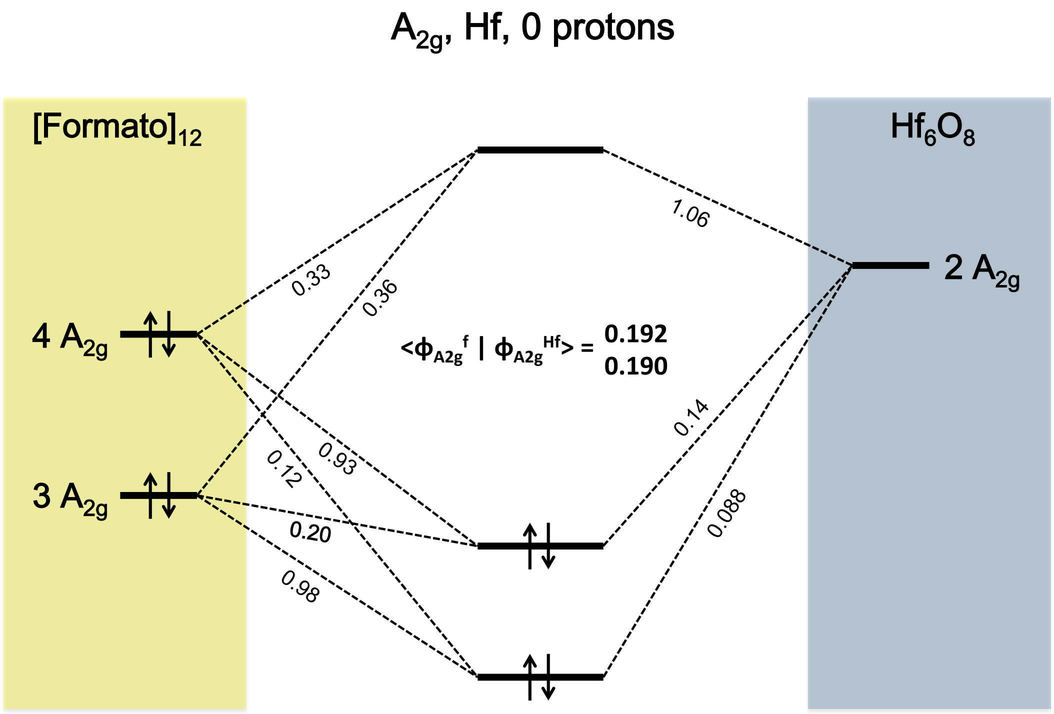

Supplementary Figure 16 *A_{2g}* MO diagram for interaction between the formato$_{12}$ fragment and the non-protonated Hf_6O_8 fragment.

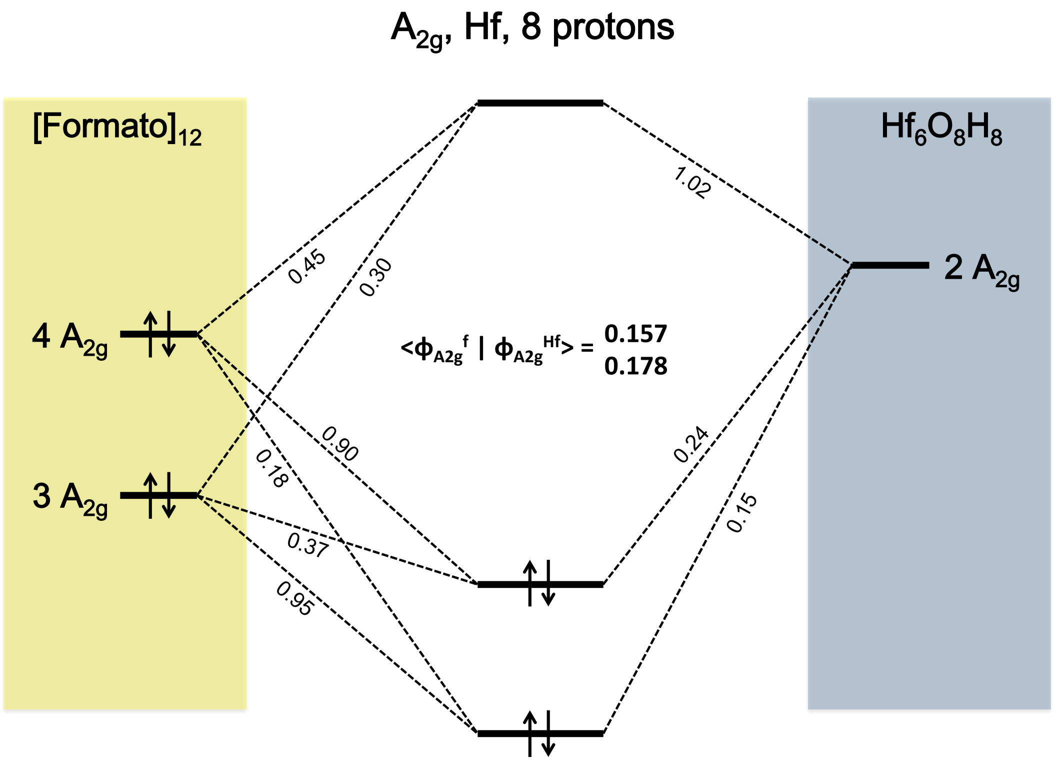

Supplementary Figure 17 *A_{2g}* MO diagram diagram for interaction between the formato$_{12}$ fragment and the 8-protonated $Hf_6O_8H_8$ fragment.

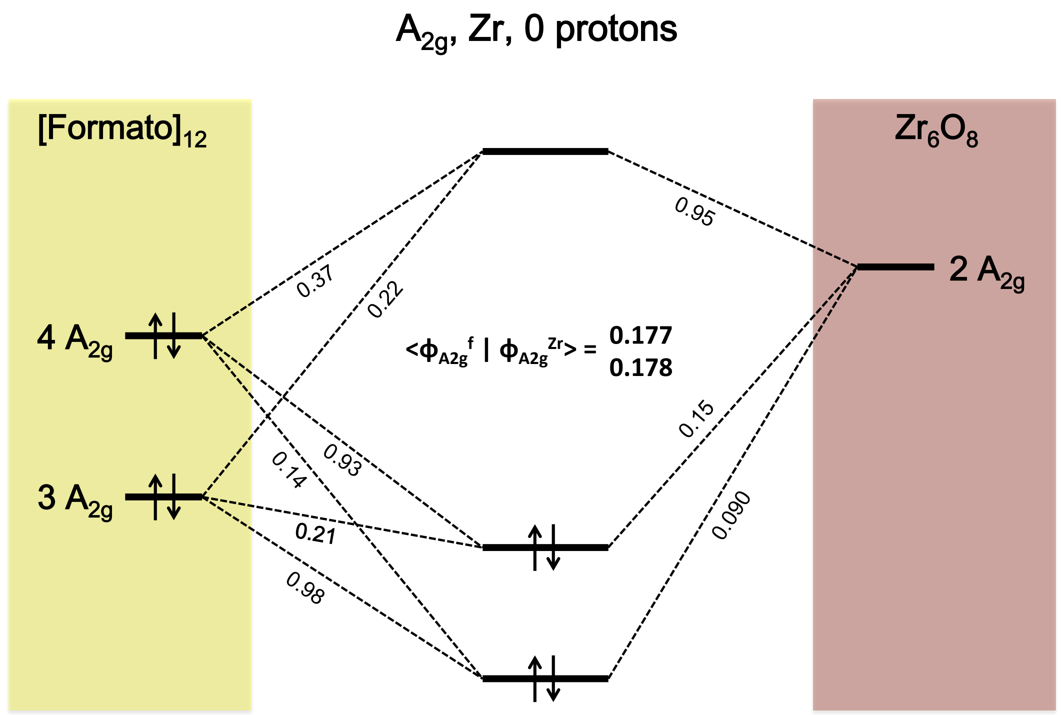

Supplementary Figure 18 A_{2g} MO diagram for interaction between the formato$_{12}$ fragment and the non-protonated Zr_6O_8 fragment.

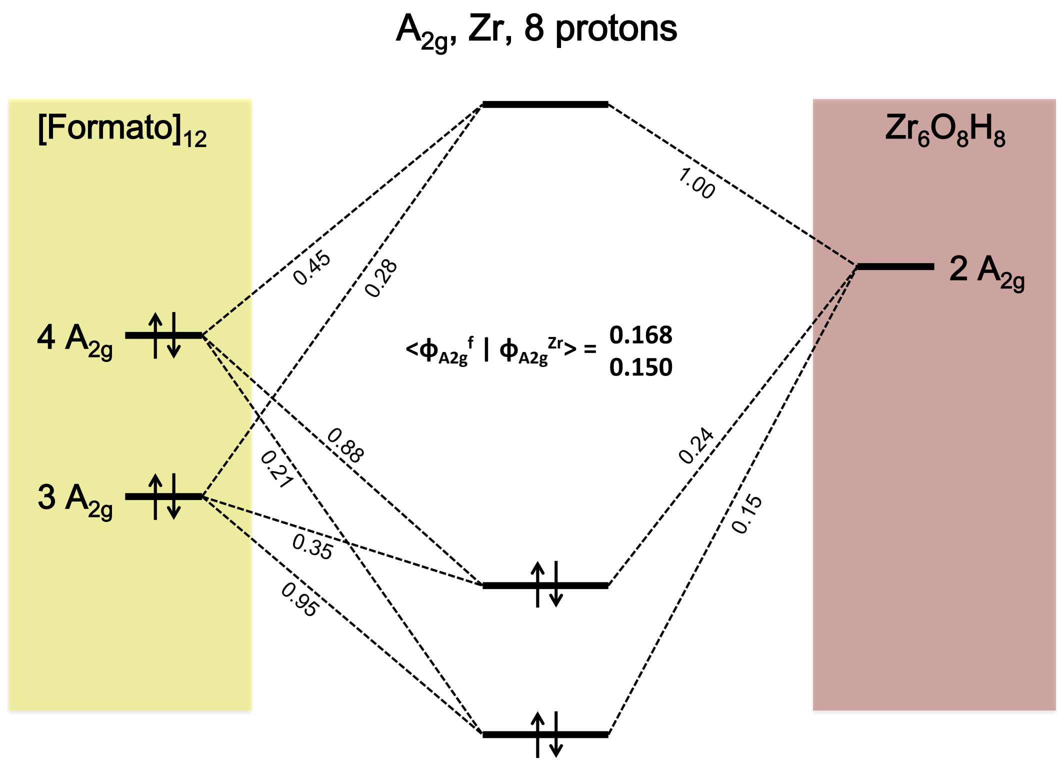

Supplementary Figure 19 A_{2g} MO diagram diagram for interaction between the formato$_{12}$ fragment and the 8-protonated $Zr_6O_8H_8$ fragment.

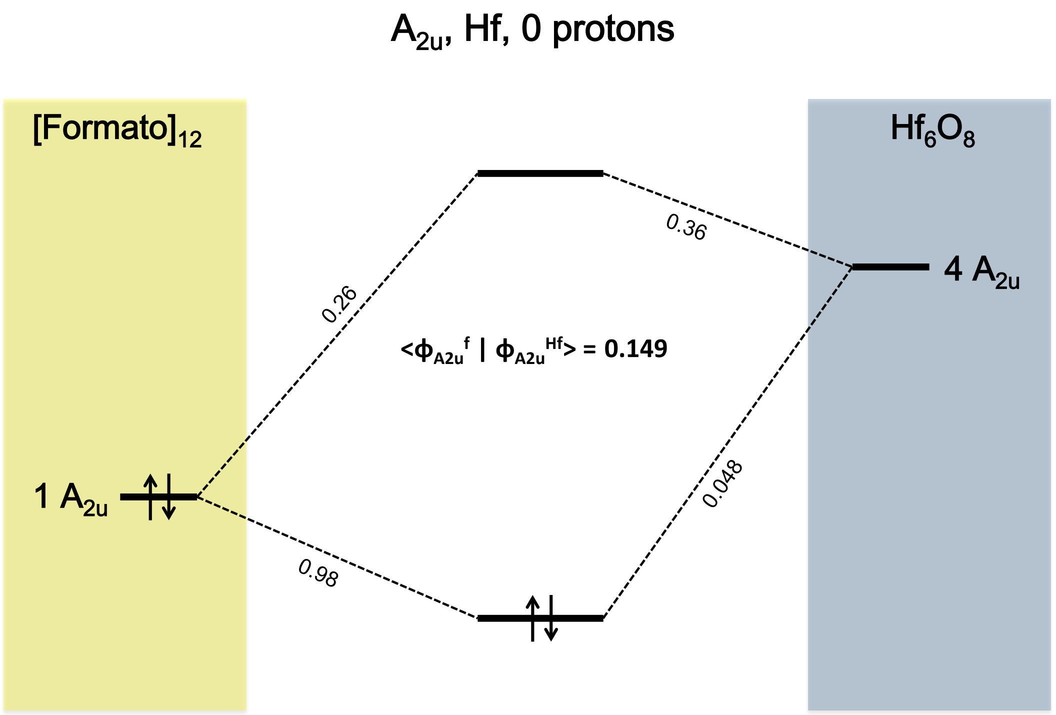

Supplementary Figure 20 A_{2u} MO diagram for interaction between the formato$_{12}$ fragment and the non-protonated Hf_6O_8 fragment.

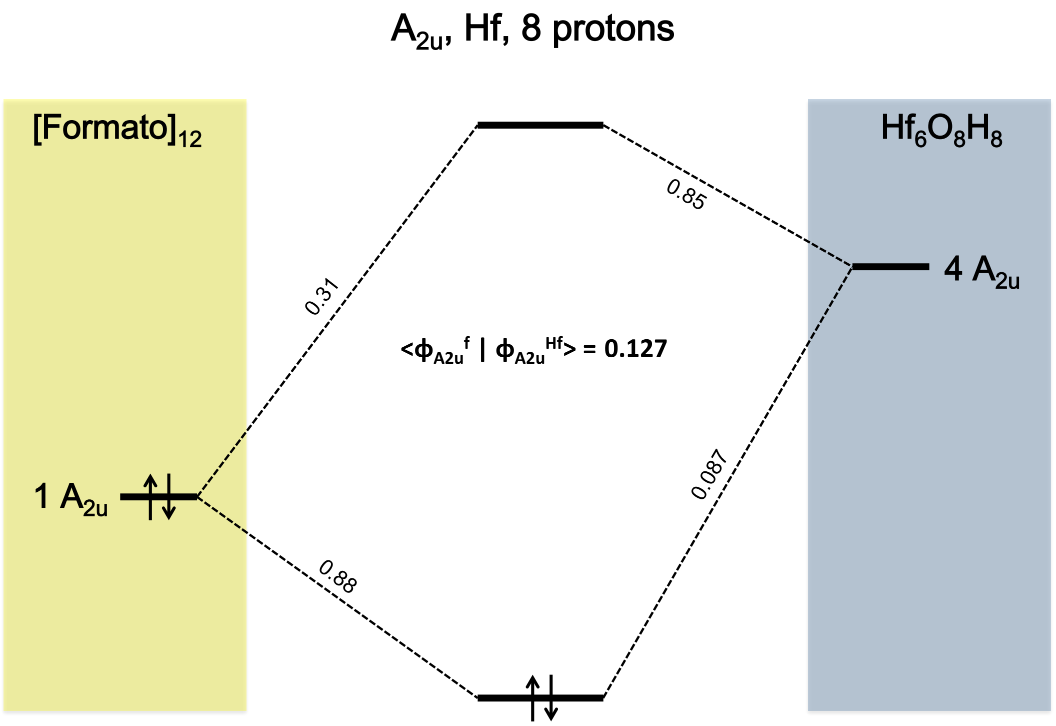

Supplementary Figure 21 A_{2u} MO diagram diagram for interaction between the formato$_{12}$ fragment and the 8-protonated $Hf_6O_8H_8$ fragment.

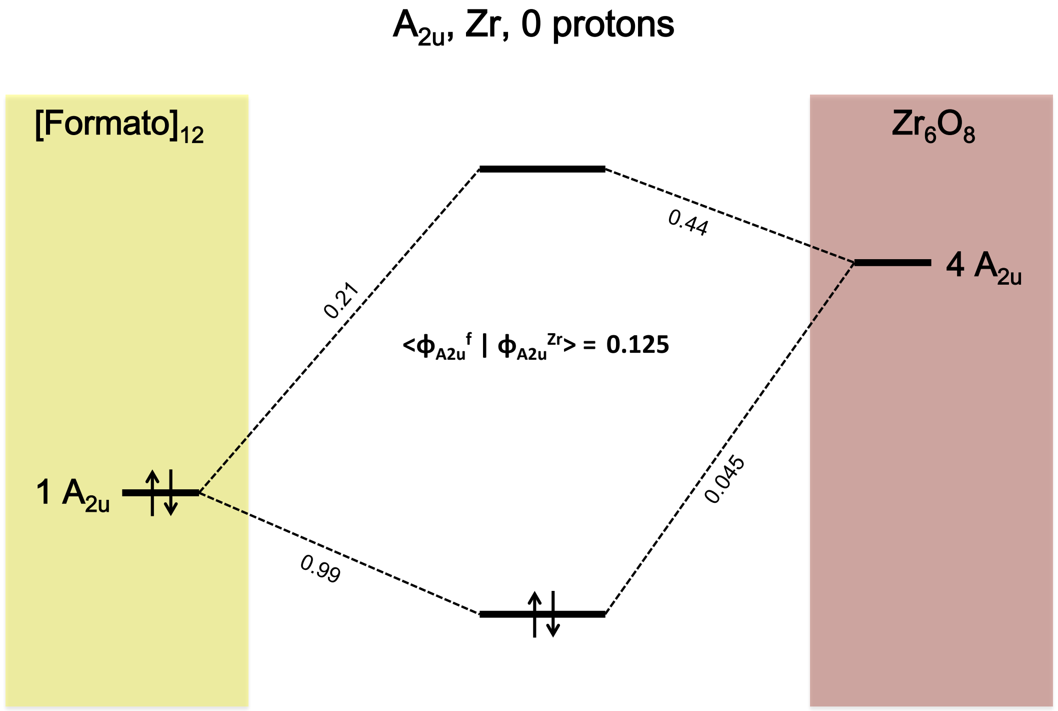

Supplementary Figure 22 A_{2u} MO diagram for interaction between the formato$_{12}$ fragment and the non-protonated Zr_6O_8 fragment.

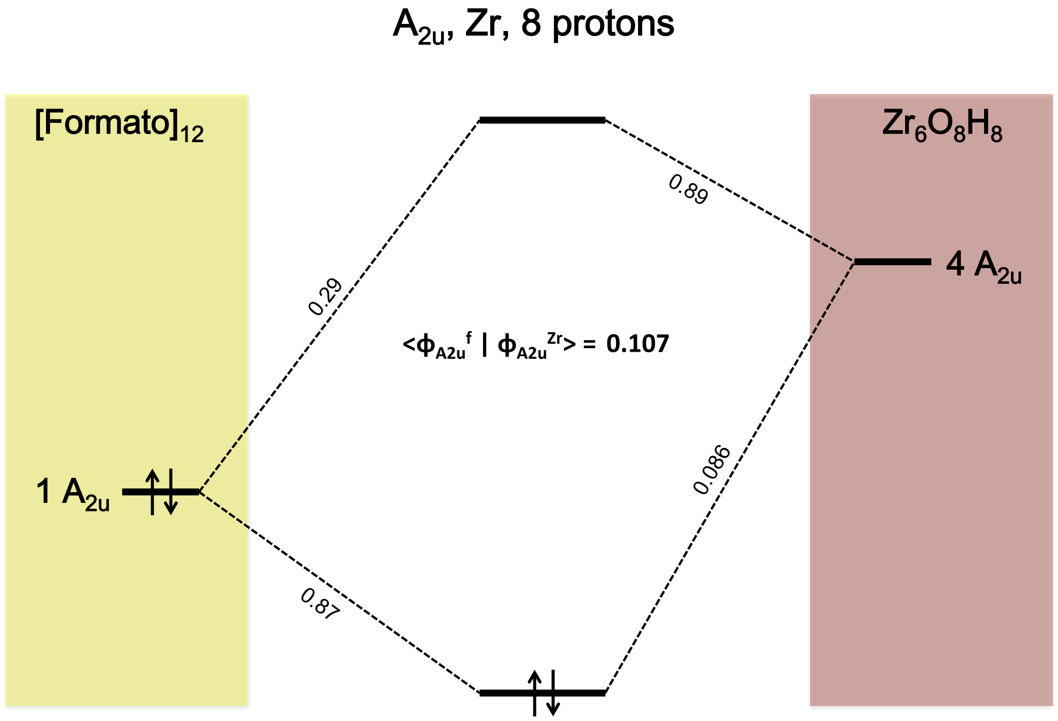

Supplementary Figure 23 A_{2u} MO diagram diagram for interaction between the formato$_{12}$ fragment and the 8-protonated $Zr_6O_8H_8$ fragment.

Supplementary Table 1: DFT Energy decomposition analysis
Energies in kcal/mol, per formato-metal bond. The total energy ΔE_{tot} contains a small energy-dispersive term, ΔE_{disp} calculated by the correction proposed by Grimme et al.[1]

Zr

	ΔE_{tot}	ΔE_{Pauli}	ΔE_{elstat}	ΔE_{orb}
**0 proton**	-302.98	44.52	-281.75	-61.83
**1 proton**	-345.10	44.12	-312.98	-72.24
**2 proton**	-388.11	43.82	-344.30	-83.56
**3 proton**	-431.99	43.59	-375.67	-95.79
**4 proton**	-476.71	43.45	-407.09	-108.86
**5 proton**	-522.23	43.50	-438.57	-122.87
**6 proton**	-568.61	43.57	-470.08	-137.75
**7 proton**	-615.81	43.68	-501.61	-153.46
**8 proton**	-663.80	43.81	-533.16	-169.96

Hf

	ΔE_{tot}	ΔE_{Pauli}	ΔE_{elstat}	ΔE_{orb}
**0 proton**	-304.84	46.07	-284.51	-62.67
**1 proton**	-346.93	45.51	-315.67	-72.97
**2 proton**	-389.88	45.03	-346.90	-84.14
**3 proton**	-433.69	44.67	-378.20	-96.21
**4 proton**	-478.28	44.38	-409.57	-109.08
**5 proton**	-523.66	44.32	-441.01	-122.89
**6 proton**	-569.86	44.30	-472.48	-137.53
**7 proton**	-616.85	44.31	-503.98	-152.96
**8 proton**	-664.59	44.35	-535.51	-169.15

Supplementary Table 2: Mulliken Populations

Zr

	0 pr.	1 pr.	2 pr.	3 pr.	4 pr.	5 pr.	6 pr.	7 pr.	8 pr.	
3 t_{2u}	0.21	0.23	0.28	0.31	0.35	0.37	0.41	0.44	0.48	***3 t_{2u}***
3 t_{2u}	0.21	0.23	0.28	0.30	0.35	0.37	0.39	0.44	0.48	***3 t_{2u}***
3 t_{2u}	0.21	0.24	0.28	0.30	0.35	0.36	0.39	0.16	0.48	***3 t_{2u}***
4 e_g	0.07	0.08	0.10	0.10	0.15	0.12	0.17	0.16	0.20	***4 e_g***
4 e_g	0.07	0.08	0.10	0.10	0.15	0.12	0.17	0.40	0.20	***4 e_g***
5 e_g	0.18	0.06	0.07	0.08	0.27	0.11	0.15	0.14	0.14	***7 t_{1u}***
5 e_g	0.18	0.22	0.23	0.27	0.27	0.36	0.15	0.28	0.14	***7 t_{1u}***
7 t_{1u}	0.06	0.22	0.23	0.27	0.09	0.36	0.36	0.28	0.14	***7 t_{1u}***
7 t_{1u}	0.06	0.08	0.18	0.10	0.09	0.11	0.36	0.07	0.44	***5 e_g***
7 t_{1u}	0.06	0.08	0.08	0.10	0.09	0.11	0.10	0.28	0.44	***5 e_g***
1 a_{2g}	0.15	0.18	0.08	0.21	0.21	0.06	0.24	0.28	0.15	***5 a_{1g}***
5 t_{2g}	0.08	0.05	0.08	0.06	0.07	0.26	0.10	0.30	0.27	***1 a_{2g}***
5 t_{2g}	0.08	0.08	0.04	0.09	0.09	0.10	0.04	0.11	0.04	***3 a_{2u}***
5 t_{2g}	0.08	0.08	0.09	0.09	0.09	0.10	0.10	0.11	0.11	***5 t_{2g}***
3 a_{2u}	0.02	0.09	0.09	0.09	0.09	0.09	0.10	0.11	0.11	***5 t_{2g}***
5 a_{1g}	0.03	0.04	0.07	0.11	0.03	0.10	0.11	0.13	0.11	***5 t_{2g}***
3 t_{1g}	0.08	0.09	0.09	0.10	0.11	0.12	0.11	0.13	0.14	***8 t_{1u}***
3 t_{1g}	0.08	0.09	0.09	0.10	0.11	0.11	0.13	0.11	0.14	***8 t_{1u}***
3 t_{1g}	0.08	0.09	0.08	0.05	0.11	0.11	0.13	0.12	0.14	***8 t_{1u}***
8 t_{1u}	0.06	0.05	0.08	0.10	0.11	0.12	0.11	0.11	0.11	***3 t_{1g}***
8 t_{1u}	0.06	0.07	0.08	0.10	0.11	0.12	0.11	0.14	0.11	***3 t_{1g}***
8 t_{1u}	0.06	0.07	0.09	0.07	0.11	0.09	0.15	0.14	0.11	***3 t_{1g}***
4 t_{2u}	0.10	0.09	0.11	0.11	0.05	0.12	0.07	0.12	0.07	***2 e_u***
4 t_{2u}	0.10	0.09	0.11	0.11	0.05	0.12	0.07	0.12	0.07	***2 e_u***
4 t_{2u}	0.10	0.10	0.11	0.07	0.11	0.07	0.12	0.10	0.15	***4 t_{2u}***
6 t_{2g}	0.07	0.08	0.10	0.07	0.11	0.07	0.12	0.14	0.15	***4 t_{2u}***
6 t_{2g}	0.07	0.08	0.04	0.11	0.11	0.15	0.17	0.15	0.15	***4 t_{2u}***
6 t_{2g}	0.07	0.08	0.04	0.12	0.13	0.11	0.17	0.15	0.19	***6 t_{2g}***
2 e_u	0.03	0.06	0.11	0.10	0.13	0.13	0.15	0.15	0.19	***6 t_{2g}***
2 e_u	0.03	0.05	0.11	0.10	0.13	0.13	0.16	0.17	0.19	***6 t_{2g}***
6 a_{1g}	0.03	0.07	0.09	0.11	0.13	0.15	0.13	0.13	0.17	***6 a_{1g}***
9 t_{1u}	0.02	0.03	0.06	0.07	0.09	0.10	0.13	0.13	0.14	***9 t_{1u}***
9 t_{1u}	0.02	0.03	0.06	0.07	0.09	0.10	0.11	0.12	0.14	***9 t_{1u}***
9 t_{1u}	0.02	0.04	0.06	0.08	0.09	0.11	0.04	0.06	0.14	***9 t_{1u}***
6 e_g	-0.02	0.00	0.00	0.02	0.02	0.04	0.04	0.05	0.07	***6 e_g***
6 e_g	-0.02	0.00	0.00	0.02	0.02	0.04	0.04	0.05	0.07	***6 e_g***
	**2.79**	**3.30**	**3.79**	**4.26**	**4.66**	**5.21**	**5.60**	**6.08**	**6.27**	

Hf

	0 pr.	1 pr.	2 pr.	3 pr.	4 pr.	5 pr.	6 pr.	7 pr.	8 pr.	
6 t_{2u}	0.21	0.23	0.27	0.30	0.34	0.36	0.39	0.42	0.44	***6 e_g***
6 t_{2u}	0.21	0.23	0.27	0.30	0.34	0.36	0.14	0.42	0.44	***6 e_g***
6 t_{2u}	0.21	0.24	0.24	0.29	0.34	0.12	0.36	0.15	0.46	***6 t_{2u}***
6 e_g	0.15	0.08	0.24	0.09	0.30	0.35	0.36	0.15	0.46	***6 t_{2u}***
6 e_g	0.15	0.08	0.28	0.09	0.30	0.11	0.40	0.15	0.46	***6 t_{2u}***
7 e_g	0.10	0.07	0.09	0.10	0.11	0.11	0.15	0.39	0.17	***6 a_{1g}***
7 e_g	0.10	0.22	0.09	0.28	0.11	0.34	0.14	0.38	0.17	***7 e_g***
10 t_{1u}	0.06	0.22	0.07	0.28	0.09	0.34	0.13	0.38	0.17	***7 e_g***
10 t_{1u}	0.06	0.07	0.08	0.09	0.09	0.10	0.10	0.16	0.14	***10 t_{1u}***

**10 t_{1u}**	0.06	0.07	0.08	0.09	0.09	0.10	0.31	0.16	0.14	**10 t_{1u}**
**2 a_{2g}**	0.16	0.18	0.19	0.21	0.12	0.08	0.07	0.07	0.14	**10 t_{1u}**
**6 a_{1g}**	0.09	0.09	0.12	0.08	0.22	0.26	0.13	0.29	0.28	**2 a_{2g}**
**8 t_{2g}**	0.09	0.09	0.05	0.09	0.09	0.11	0.32	0.12	0.04	**4 a_{2u}**
**8 t_{2g}**	0.09	0.09	0.10	0.09	0.09	0.09	0.11	0.10	0.10	**8 t_{2g}**
**8 t_{2g}**	0.09	0.09	0.09	0.10	0.09	0.09	0.13	0.10	0.10	**8 t_{2g}**
**4 a_{2u}**	0.02	0.08	0.09	0.12	0.04	0.10	0.10	0.13	0.10	**8 t_{2g}**
**5 t_{1g}**	0.08	0.08	0.09	0.11	0.11	0.12	0.10	0.13	0.11	**5 t_{1g}**
**5 t_{1g}**	0.08	0.07	0.09	0.11	0.11	0.12	0.07	0.12	0.11	**5 t_{1g}**
**5 t_{1g}**	0.08	0.09	0.08	0.06	0.11	0.12	0.11	0.12	0.11	**5 t_{1g}**
**7 a_{1g}**	0.02	0.06	0.09	0.11	0.13	0.13	0.13	0.15	0.14	**11 t_{1u}**
**11 t_{1u}**	0.06	0.07	0.08	0.11	0.13	0.13	0.13	0.15	0.14	**11 t_{1u}**
**11 t_{1u}**	0.06	0.07	0.08	0.09	0.13	0.10	0.14	0.12	0.14	**11 t_{1u}**
**11 t_{1u}**	0.06	0.05	0.07	0.10	0.05	0.12	0.12	0.12	0.07	**3 e_u**
**7 t_{2u}**	0.10	0.09	0.11	0.10	0.05	0.12	0.14	0.12	0.07	**3 e_u**
**7 t_{2u}**	0.10	0.09	0.11	0.09	0.12	0.15	0.08	0.10	0.16	**7 t_{2u}**
**7 t_{2u}**	0.10	0.10	0.11	0.08	0.12	0.07	0.13	0.10	0.16	**7 t_{2u}**
**9 t_{2g}**	0.07	0.09	0.12	0.08	0.12	0.07	0.12	0.14	0.16	**7 t_{2u}**
**9 t_{2g}**	0.07	0.09	0.11	0.11	0.13	0.17	0.18	0.21	0.20	**9 t_{2g}**
**9 t_{2g}**	0.07	0.08	0.11	0.13	0.13	0.12	0.18	0.15	0.20	**9 t_{2g}**
**12 t_{1u}**	0.06	0.08	0.05	0.09	0.13	0.13	0.10	0.16	0.20	**9 t_{2g}**
**12 t_{1u}**	0.06	0.08	0.05	0.09	0.14	0.13	0.14	0.16	0.22	**7 a_{1g}**
**12 t_{1u}**	0.06	0.08	0.09	0.11	0.12	0.12	0.13	0.14	0.16	**12 t_{1u}**
**8 e_g**	0.03	0.05	0.09	0.11	0.12	0.12	0.15	0.14	0.16	**12 t_{1u}**
**8 e_g**	0.03	0.05	0.09	0.11	0.12	0.13	0.15	0.15	0.16	**12 t_{1u}**
**3 e_u**	0.03	0.05	0.05	0.07	0.07	0.09	0.08	0.10	0.09	**8 e_g**
**3 e_u**	0.03	0.05	0.05	0.07	0.07	0.09	0.10	0.10	0.09	**8 e_g**
	**3.10**	**3.60**	**4.07**	**4.53**	**4.97**	**5.37**	**5.82**	**6.25**	**6.66**	

Supplementary Table 3: Coordinates of all calculated systems

Zr, 0 protons

O	3.73236000	-1.83921000	1.74103000 f=formato
O	-1.83921000	-0.25030000	1.74103000 f=formato
O	1.74103000	3.73236000	-1.83921000 f=formato
O	-1.83921000	1.74103000	-0.25030000 f=formato
O	-1.83921000	1.74103000	3.73236000 f=formato
O	1.74103000	3.73236000	5.32127000 f=formato
O	5.32127000	1.74103000	-0.25030000 f=formato
O	1.74103000	-0.25030000	5.32127000 f=formato
O	1.74103000	-0.25030000	-1.83921000 f=formato
O	5.32127000	1.74103000	3.73236000 f=formato
C	4.79647000	1.74103000	4.79647000 f=formato
O	3.73236000	5.32127000	1.74103000 f=formato
O	1.74103000	5.32127000	-0.25030000 f=formato
C	4.79647000	1.74103000	-1.31441000 f=formato
O	1.74103000	-1.83921000	3.73236000 f=formato
O	5.32127000	-0.25030000	1.74103000 f=formato
C	1.74103000	4.79647000	4.79647000 f=formato
C	1.74103000	-1.31441000	4.79647000 f=formato
O	-0.25030000	5.32127000	1.74103000 f=formato
C	4.79647000	-1.31441000	1.74103000 f=formato
O	3.73236000	1.74103000	-1.83921000 f=formato
C	-1.31441000	1.74103000	-1.31441000 f=formato
O	3.73236000	1.74103000	5.32127000 f=formato
C	-1.31441000	-1.31441000	1.74103000 f=formato
C	1.74103000	4.79647000	-1.31441000 f=formato
O	-0.25030000	-1.83921000	1.74103000 f=formato
C	-1.31441000	4.79647000	1.74103000 f=formato
O	-0.25030000	1.74103000	5.32127000 f=formato
C	-1.31441000	1.74103000	4.79647000 f=formato
H	1.74103000	-2.07809000	-2.07809000 f=formato
H	5.56015000	-2.07809000	1.74103000 f=formato
H	1.74103000	-2.07809000	5.56015000 f=formato
H	-2.07809000	-2.07809000	1.74103000 f=formato
O	-0.25030000	1.74103000	-1.83921000 f=formato
C	1.74103000	-1.31441000	-1.31441000 f=formato
O	1.74103000	5.32127000	3.73236000 f=formato
O	5.32127000	3.73236000	1.74103000 f=formato
H	1.74103000	5.56015000	5.56015000 f=formato
H	-2.07809000	1.74103000	5.56015000 f=formato
H	-2.07809000	5.56015000	1.74103000 f=formato
H	1.74103000	5.56015000	-2.07809000 f=formato
H	5.56015000	1.74103000	-2.07809000 f=formato
H	5.56015000	5.56015000	1.74103000 f=formato
H	5.56015000	1.74103000	5.56015000 f=formato
H	-2.07809000	1.74103000	-2.07809000 f=formato
O	-1.83921000	3.73236000	1.74103000 f=formato
C	4.79647000	4.79647000	1.74103000 f=formato
O	1.74103000	-1.83921000	-0.25030000 f=formato
O	0.57735000	2.90471000	2.90471000 f=Zr0
O	2.90471000	2.90471000	2.90471000 f=Zr0
O	2.90471000	0.57735000	0.57735000 f=Zr0
O	0.57735000	2.90471000	0.57735000 f=Zr0
O	0.57735000	0.57735000	2.90471000 f=Zr0
Zr	1.74103000	4.23849000	1.74103000 f=Zr0
Zr	1.74103000	1.74103000	-0.75643000 f=Zr0
Zr	1.74103000	-0.75643000	1.74103000 f=Zr0
Zr	1.74103000	1.74103000	4.23849000 f=Zr0
Zr	4.23849000	1.74103000	1.74103000 f=Zr0

Zr	-0.75643000	1.74103000	1.74103000	f=Zr0
O	0.57735000	0.57735000	0.57735000	f=Zr0
O	2.90471000	2.90471000	0.57735000	f=Zr0
O	2.90471000	0.57735000	2.90471000	f=Zr0

Zr, 1 protons

O	3.73236000	-1.83921000	1.74103000	f=formato
O	-1.83921000	-0.25030000	1.74103000	f=formato
O	1.74103000	3.73236000	-1.83921000	f=formato
O	-1.83921000	1.74103000	-0.25030000	f=formato
O	-1.83921000	1.74103000	3.73236000	f=formato
O	1.74103000	3.73236000	5.32127000	f=formato
O	5.32127000	1.74103000	-0.25030000	f=formato
O	1.74103000	-0.25030000	5.32127000	f=formato
O	1.74103000	-0.25030000	-1.83921000	f=formato
O	5.32127000	1.74103000	3.73236000	f=formato
C	4.79647000	1.74103000	4.79647000	f=formato
O	3.73236000	5.32127000	1.74103000	f=formato
O	1.74103000	5.32127000	-0.25030000	f=formato
C	4.79647000	1.74103000	-1.31441000	f=formato
O	1.74103000	-1.83921000	3.73236000	f=formato
O	5.32127000	-0.25030000	1.74103000	f=formato
C	1.74103000	4.79647000	4.79647000	f=formato
C	1.74103000	-1.31441000	4.79647000	f=formato
O	-0.25030000	5.32127000	1.74103000	f=formato
C	4.79647000	-1.31441000	1.74103000	f=formato
O	3.73236000	1.74103000	-1.83921000	f=formato
C	-1.31441000	1.74103000	-1.31441000	f=formato
O	3.73236000	1.74103000	5.32127000	f=formato
C	-1.31441000	-1.31441000	1.74103000	f=formato
C	1.74103000	4.79647000	-1.31441000	f=formato
O	-0.25030000	-1.83921000	1.74103000	f=formato
C	-1.31441000	4.79647000	1.74103000	f=formato
O	-0.25030000	1.74103000	5.32127000	f=formato
C	-1.31441000	1.74103000	4.79647000	f=formato
H	1.74103000	-2.07809000	-2.07809000	f=formato
H	5.56015000	-2.07809000	1.74103000	f=formato
H	1.74103000	-2.07809000	5.56015000	f=formato
H	-2.07809000	-2.07809000	1.74103000	f=formato
O	-0.25030000	1.74103000	-1.83921000	f=formato
C	1.74103000	-1.31441000	-1.31441000	f=formato
O	1.74103000	5.32127000	3.73236000	f=formato
O	5.32127000	3.73236000	1.74103000	f=formato
H	1.74103000	5.56015000	5.56015000	f=formato
H	-2.07809000	1.74103000	5.56015000	f=formato
H	-2.07809000	5.56015000	1.74103000	f=formato
H	1.74103000	5.56015000	-2.07809000	f=formato
H	5.56015000	1.74103000	-2.07809000	f=formato
H	5.56015000	5.56015000	1.74103000	f=formato
H	5.56015000	1.74103000	5.56015000	f=formato
H	-2.07809000	1.74103000	-2.07809000	f=formato
O	-1.83921000	3.73236000	1.74103000	f=formato
C	4.79647000	4.79647000	1.74103000	f=formato
O	1.74103000	-1.83921000	-0.25030000	f=formato
O	0.57735000	2.90471000	2.90471000	f=Zr1
O	2.90471000	2.90471000	2.90471000	f=Zr1
H	3.48206000	0.00000000	0.00000000	f=Zr1
O	2.90471000	0.57735000	0.57735000	f=Zr1
O	0.57735000	2.90471000	0.57735000	f=Zr1

O	0.57735000	0.57735000	2.90471000	f=Zr1
Zr	1.74103000	4.23849000	1.74103000	f=Zr1
Zr	1.74103000	1.74103000	-0.75643000	f=Zr1
Zr	1.74103000	-0.75643000	1.74103000	f=Zr1
Zr	1.74103000	1.74103000	4.23849000	f=Zr1
Zr	4.23849000	1.74103000	1.74103000	f=Zr1
Zr	-0.75643000	1.74103000	1.74103000	f=Zr1
O	0.57735000	0.57735000	0.57735000	f=Zr1
O	2.90471000	2.90471000	0.57735000	f=Zr1
O	2.90471000	0.57735000	2.90471000	f=Zr1

Zr, 2 protons

O	3.73236000	-1.83921000	1.74103000	f=formato
O	-1.83921000	-0.25030000	1.74103000	f=formato
O	1.74103000	3.73236000	-1.83921000	f=formato
O	-1.83921000	1.74103000	-0.25030000	f=formato
O	-1.83921000	1.74103000	3.73236000	f=formato
O	1.74103000	3.73236000	5.32127000	f=formato
O	5.32127000	1.74103000	-0.25030000	f=formato
O	1.74103000	-0.25030000	5.32127000	f=formato
O	1.74103000	-0.25030000	-1.83921000	f=formato
O	5.32127000	1.74103000	3.73236000	f=formato
C	4.79647000	1.74103000	4.79647000	f=formato
O	3.73236000	5.32127000	1.74103000	f=formato
O	1.74103000	5.32127000	-0.25030000	f=formato
C	4.79647000	1.74103000	-1.31441000	f=formato
O	1.74103000	-1.83921000	3.73236000	f=formato
O	5.32127000	-0.25030000	1.74103000	f=formato
C	1.74103000	4.79647000	4.79647000	f=formato
C	1.74103000	-1.31441000	4.79647000	f=formato
O	-0.25030000	5.32127000	1.74103000	f=formato
C	4.79647000	-1.31441000	1.74103000	f=formato
O	3.73236000	1.74103000	-1.83921000	f=formato
C	-1.31441000	1.74103000	-1.31441000	f=formato
O	3.73236000	1.74103000	5.32127000	f=formato
C	-1.31441000	-1.31441000	1.74103000	f=formato
C	1.74103000	4.79647000	-1.31441000	f=formato
O	-0.25030000	-1.83921000	1.74103000	f=formato
C	-1.31441000	4.79647000	1.74103000	f=formato
O	-0.25030000	1.74103000	5.32127000	f=formato
C	-1.31441000	1.74103000	4.79647000	f=formato
H	1.74103000	-2.07809000	-2.07809000	f=formato
H	5.56015000	-2.07809000	1.74103000	f=formato
H	1.74103000	-2.07809000	5.56015000	f=formato
H	-2.07809000	-2.07809000	1.74103000	f=formato
O	-0.25030000	1.74103000	-1.83921000	f=formato
C	1.74103000	-1.31441000	-1.31441000	f=formato
O	1.74103000	5.32127000	3.73236000	f=formato
O	5.32127000	3.73236000	1.74103000	f=formato
H	1.74103000	5.56015000	5.56015000	f=formato
H	-2.07809000	1.74103000	5.56015000	f=formato
H	-2.07809000	5.56015000	1.74103000	f=formato
H	1.74103000	5.56015000	-2.07809000	f=formato
H	5.56015000	1.74103000	-2.07809000	f=formato
H	5.56015000	5.56015000	1.74103000	f=formato
H	5.56015000	1.74103000	5.56015000	f=formato
H	-2.07809000	1.74103000	-2.07809000	f=formato
O	-1.83921000	3.73236000	1.74103000	f=formato
C	4.79647000	4.79647000	1.74103000	f=formato
O	1.74103000	-1.83921000	-0.25030000	f=formato

O	0.57735000	2.90471000	2.90471000	f=Zr2
O	2.90471000	2.90471000	2.90471000	f=Zr2
H	3.48206000	0.00000000	0.00000000	f=Zr2
O	2.90471000	0.57735000	0.57735000	f=Zr2
O	0.57735000	2.90471000	0.57735000	f=Zr2
H	0.00000000	3.48206000	3.48206000	f=Zr2
O	0.57735000	0.57735000	2.90471000	f=Zr2
Zr	1.74103000	4.23849000	1.74103000	f=Zr2
Zr	1.74103000	1.74103000	-0.75643000	f=Zr2
Zr	1.74103000	-0.75643000	1.74103000	f=Zr2
Zr	1.74103000	1.74103000	4.23849000	f=Zr2
Zr	4.23849000	1.74103000	1.74103000	f=Zr2
Zr	-0.75643000	1.74103000	1.74103000	f=Zr2
O	0.57735000	0.57735000	0.57735000	f=Zr2
O	2.90471000	2.90471000	0.57735000	f=Zr2
O	2.90471000	0.57735000	2.90471000	f=Zr2

Zr, 3 protons

O	3.73236000	-1.83921000	1.74103000	f=formato
O	-1.83921000	-0.25030000	1.74103000	f=formato
O	1.74103000	3.73236000	-1.83921000	f=formato
O	-1.83921000	1.74103000	-0.25030000	f=formato
O	-1.83921000	1.74103000	3.73236000	f=formato
O	1.74103000	3.73236000	5.32127000	f=formato
O	5.32127000	1.74103000	-0.25030000	f=formato
O	1.74103000	-0.25030000	5.32127000	f=formato
O	1.74103000	-0.25030000	-1.83921000	f=formato
O	5.32127000	1.74103000	3.73236000	f=formato
C	4.79647000	1.74103000	4.79647000	f=formato
O	3.73236000	5.32127000	1.74103000	f=formato
O	1.74103000	5.32127000	-0.25030000	f=formato
C	4.79647000	1.74103000	-1.31441000	f=formato
O	1.74103000	-1.83921000	3.73236000	f=formato
O	5.32127000	-0.25030000	1.74103000	f=formato
C	1.74103000	4.79647000	4.79647000	f=formato
C	1.74103000	-1.31441000	4.79647000	f=formato
O	-0.25030000	5.32127000	1.74103000	f=formato
C	4.79647000	-1.31441000	1.74103000	f=formato
O	3.73236000	1.74103000	-1.83921000	f=formato
C	-1.31441000	1.74103000	-1.31441000	f=formato
O	3.73236000	1.74103000	5.32127000	f=formato
C	-1.31441000	-1.31441000	1.74103000	f=formato
C	1.74103000	4.79647000	-1.31441000	f=formato
O	-0.25030000	-1.83921000	1.74103000	f=formato
C	-1.31441000	4.79647000	1.74103000	f=formato
O	-0.25030000	1.74103000	5.32127000	f=formato
C	-1.31441000	1.74103000	4.79647000	f=formato
H	1.74103000	-2.07809000	-2.07809000	f=formato
H	5.56015000	-2.07809000	1.74103000	f=formato
H	1.74103000	-2.07809000	5.56015000	f=formato
H	-2.07809000	-2.07809000	1.74103000	f=formato
O	-0.25030000	1.74103000	-1.83921000	f=formato
C	1.74103000	-1.31441000	-1.31441000	f=formato
O	1.74103000	5.32127000	3.73236000	f=formato
O	5.32127000	3.73236000	1.74103000	f=formato
H	1.74103000	5.56015000	5.56015000	f=formato
H	-2.07809000	1.74103000	5.56015000	f=formato
H	-2.07809000	5.56015000	1.74103000	f=formato
H	1.74103000	5.56015000	-2.07809000	f=formato
H	5.56015000	1.74103000	-2.07809000	f=formato

H	5.56015000	5.56015000	1.74103000	f=formato
H	5.56015000	1.74103000	5.56015000	f=formato
H	-2.07809000	1.74103000	-2.07809000	f=formato
O	-1.83921000	3.73236000	1.74103000	f=formato
C	4.79647000	4.79647000	1.74103000	f=formato
O	1.74103000	-1.83921000	-0.25030000	f=formato
O	0.57735000	2.90471000	2.90471000	f=Zr3
O	2.90471000	2.90471000	2.90471000	f=Zr3
H	3.48206000	3.48206000	0.00000000	f=Zr3
O	2.90471000	0.57735000	0.57735000	f=Zr3
H	3.48206000	0.00000000	3.48206000	f=Zr3
O	0.57735000	2.90471000	0.57735000	f=Zr3
H	0.00000000	3.48206000	3.48206000	f=Zr3
O	0.57735000	0.57735000	2.90471000	f=Zr3
Zr	1.74103000	4.23849000	1.74103000	f=Zr3
Zr	1.74103000	1.74103000	-0.75643000	f=Zr3
Zr	1.74103000	-0.75643000	1.74103000	f=Zr3
Zr	1.74103000	1.74103000	4.23849000	f=Zr3
Zr	4.23849000	1.74103000	1.74103000	f=Zr3
Zr	-0.75643000	1.74103000	1.74103000	f=Zr3
O	0.57735000	0.57735000	0.57735000	f=Zr3
O	2.90471000	2.90471000	0.57735000	f=Zr3
O	2.90471000	0.57735000	2.90471000	f=Zr3

Zr, 4 protons

O	3.73236000	-1.83921000	1.74103000	f=formato
O	-1.83921000	-0.25030000	1.74103000	f=formato
O	1.74103000	3.73236000	-1.83921000	f=formato
O	-1.83921000	1.74103000	-0.25030000	f=formato
O	-1.83921000	1.74103000	3.73236000	f=formato
O	1.74103000	3.73236000	5.32127000	f=formato
O	5.32127000	1.74103000	-0.25030000	f=formato
O	1.74103000	-0.25030000	5.32127000	f=formato
O	1.74103000	-0.25030000	-1.83921000	f=formato
O	5.32127000	1.74103000	3.73236000	f=formato
C	4.79647000	1.74103000	4.79647000	f=formato
O	3.73236000	5.32127000	1.74103000	f=formato
O	1.74103000	5.32127000	-0.25030000	f=formato
C	4.79647000	1.74103000	-1.31441000	f=formato
O	1.74103000	-1.83921000	3.73236000	f=formato
O	5.32127000	-0.25030000	1.74103000	f=formato
C	1.74103000	4.79647000	4.79647000	f=formato
C	1.74103000	-1.31441000	4.79647000	f=formato
O	-0.25030000	5.32127000	1.74103000	f=formato
C	4.79647000	-1.31441000	1.74103000	f=formato
O	3.73236000	1.74103000	-1.83921000	f=formato
C	-1.31441000	1.74103000	-1.31441000	f=formato
O	3.73236000	1.74103000	5.32127000	f=formato
C	-1.31441000	-1.31441000	1.74103000	f=formato
C	1.74103000	4.79647000	-1.31441000	f=formato
O	-0.25030000	-1.83921000	1.74103000	f=formato
C	-1.31441000	4.79647000	1.74103000	f=formato
O	-0.25030000	1.74103000	5.32127000	f=formato
C	-1.31441000	1.74103000	4.79647000	f=formato
H	1.74103000	-2.07809000	-2.07809000	f=formato
H	5.56015000	-2.07809000	1.74103000	f=formato
H	1.74103000	-2.07809000	5.56015000	f=formato
H	-2.07809000	-2.07809000	1.74103000	f=formato
O	-0.25030000	1.74103000	-1.83921000	f=formato

C	1.74103000	-1.31441000	-1.31441000	f=formato
O	1.74103000	5.32127000	3.73236000	f=formato
O	5.32127000	3.73236000	1.74103000	f=formato
H	1.74103000	5.56015000	5.56015000	f=formato
H	-2.07809000	1.74103000	5.56015000	f=formato
H	-2.07809000	5.56015000	1.74103000	f=formato
H	1.74103000	5.56015000	-2.07809000	f=formato
H	5.56015000	1.74103000	-2.07809000	f=formato
H	5.56015000	5.56015000	1.74103000	f=formato
H	5.56015000	1.74103000	5.56015000	f=formato
H	-2.07809000	1.74103000	-2.07809000	f=formato
O	-1.83921000	3.73236000	1.74103000	f=formato
C	4.79647000	4.79647000	1.74103000	f=formato
O	1.74103000	-1.83921000	-0.25030000	f=formato
O	0.57735000	2.90471000	2.90471000	f=Zr4
O	2.90471000	2.90471000	2.90471000	f=Zr4
H	3.48206000	3.48206000	0.00000000	f=Zr4
O	2.90471000	0.57735000	0.57735000	f=Zr4
H	3.48206000	0.00000000	3.48206000	f=Zr4
O	0.57735000	2.90471000	0.57735000	f=Zr4
H	0.00000000	3.48206000	3.48206000	f=Zr4
O	0.57735000	0.57735000	2.90471000	f=Zr4
H	0.00000000	0.00000000	0.00000000	f=Zr4
Zr	1.74103000	4.23849000	1.74103000	f=Zr4
Zr	1.74103000	1.74103000	-0.75643000	f=Zr4
Zr	1.74103000	-0.75643000	1.74103000	f=Zr4
Zr	1.74103000	1.74103000	4.23849000	f=Zr4
Zr	4.23849000	1.74103000	1.74103000	f=Zr4
Zr	-0.75643000	1.74103000	1.74103000	f=Zr4
O	0.57735000	0.57735000	0.57735000	f=Zr4
O	2.90471000	2.90471000	0.57735000	f=Zr4
O	2.90471000	0.57735000	2.90471000	f=Zr4

Zr, 5 protons

O	3.73236000	-1.83921000	1.74103000	f=formato
O	-1.83921000	-0.25030000	1.74103000	f=formato
O	1.74103000	3.73236000	-1.83921000	f=formato
O	-1.83921000	1.74103000	-0.25030000	f=formato
O	-1.83921000	1.74103000	3.73236000	f=formato
O	1.74103000	3.73236000	5.32127000	f=formato
O	5.32127000	1.74103000	-0.25030000	f=formato
O	1.74103000	-0.25030000	5.32127000	f=formato
O	1.74103000	-0.25030000	-1.83921000	f=formato
O	5.32127000	1.74103000	3.73236000	f=formato
C	4.79647000	1.74103000	4.79647000	f=formato
O	3.73236000	5.32127000	1.74103000	f=formato
O	1.74103000	5.32127000	-0.25030000	f=formato
C	4.79647000	1.74103000	-1.31441000	f=formato
O	1.74103000	-1.83921000	3.73236000	f=formato
O	5.32127000	-0.25030000	1.74103000	f=formato
C	1.74103000	4.79647000	4.79647000	f=formato
C	1.74103000	-1.31441000	4.79647000	f=formato
O	-0.25030000	5.32127000	1.74103000	f=formato
C	4.79647000	-1.31441000	1.74103000	f=formato
O	3.73236000	1.74103000	-1.83921000	f=formato
C	-1.31441000	1.74103000	-1.31441000	f=formato
O	3.73236000	1.74103000	5.32127000	f=formato
C	-1.31441000	-1.31441000	1.74103000	f=formato
C	1.74103000	4.79647000	-1.31441000	f=formato

O	-0.25030000	-1.83921000	1.74103000	f=formato
C	-1.31441000	4.79647000	1.74103000	f=formato
O	-0.25030000	1.74103000	5.32127000	f=formato
C	-1.31441000	1.74103000	4.79647000	f=formato
H	1.74103000	-2.07809000	-2.07809000	f=formato
H	5.56015000	-2.07809000	1.74103000	f=formato
H	1.74103000	-2.07809000	5.56015000	f=formato
H	-2.07809000	-2.07809000	1.74103000	f=formato
O	-0.25030000	1.74103000	-1.83921000	f=formato
C	1.74103000	-1.31441000	-1.31441000	f=formato
O	1.74103000	5.32127000	3.73236000	f=formato
O	5.32127000	3.73236000	1.74103000	f=formato
H	1.74103000	5.56015000	5.56015000	f=formato
H	-2.07809000	1.74103000	5.56015000	f=formato
H	-2.07809000	5.56015000	1.74103000	f=formato
H	1.74103000	5.56015000	-2.07809000	f=formato
H	5.56015000	1.74103000	-2.07809000	f=formato
H	5.56015000	5.56015000	1.74103000	f=formato
H	5.56015000	1.74103000	5.56015000	f=formato
H	-2.07809000	1.74103000	-2.07809000	f=formato
O	-1.83921000	3.73236000	1.74103000	f=formato
C	4.79647000	4.79647000	1.74103000	f=formato
O	1.74103000	-1.83921000	-0.25030000	f=formato
O	0.57735000	2.90471000	2.90471000	f=Zr5
O	2.90471000	2.90471000	2.90471000	f=Zr5
H	3.48206000	3.48206000	0.00000000	f=Zr5
O	2.90471000	0.57735000	0.57735000	f=Zr5
H	3.48206000	0.00000000	3.48206000	f=Zr5
O	0.57735000	2.90471000	0.57735000	f=Zr5
H	0.00000000	3.48206000	3.48206000	f=Zr5
O	0.57735000	0.57735000	2.90471000	f=Zr5
H	0.00000000	3.48206000	0.00000000	f=Zr5
H	0.00000000	0.00000000	0.00000000	f=Zr5
Zr	1.74103000	4.23849000	1.74103000	f=Zr5
Zr	1.74103000	1.74103000	-0.75643000	f=Zr5
Zr	1.74103000	-0.75643000	1.74103000	f=Zr5
Zr	1.74103000	1.74103000	4.23849000	f=Zr5
Zr	4.23849000	1.74103000	1.74103000	f=Zr5
Zr	-0.75643000	1.74103000	1.74103000	f=Zr5
O	0.57735000	0.57735000	0.57735000	f=Zr5
O	2.90471000	2.90471000	0.57735000	f=Zr5
O	2.90471000	0.57735000	2.90471000	f=Zr5

Zr, 6 protons

O	3.73236000	-1.83921000	1.74103000	f=formato
O	-1.83921000	-0.25030000	1.74103000	f=formato
O	1.74103000	3.73236000	-1.83921000	f=formato
O	-1.83921000	1.74103000	-0.25030000	f=formato
O	-1.83921000	1.74103000	3.73236000	f=formato
O	1.74103000	3.73236000	5.32127000	f=formato
O	5.32127000	1.74103000	-0.25030000	f=formato
O	1.74103000	-0.25030000	5.32127000	f=formato
O	1.74103000	-0.25030000	-1.83921000	f=formato
O	5.32127000	1.74103000	3.73236000	f=formato
C	4.79647000	1.74103000	4.79647000	f=formato
O	3.73236000	5.32127000	1.74103000	f=formato
O	1.74103000	5.32127000	-0.25030000	f=formato
C	4.79647000	1.74103000	-1.31441000	f=formato
O	1.74103000	-1.83921000	3.73236000	f=formato

O	5.32127000	-0.25030000	1.74103000	f=formato
C	1.74103000	4.79647000	4.79647000	f=formato
C	1.74103000	-1.31441000	4.79647000	f=formato
O	-0.25030000	5.32127000	1.74103000	f=formato
C	4.79647000	-1.31441000	1.74103000	f=formato
O	3.73236000	1.74103000	-1.83921000	f=formato
C	-1.31441000	1.74103000	-1.31441000	f=formato
O	3.73236000	1.74103000	5.32127000	f=formato
C	-1.31441000	-1.31441000	1.74103000	f=formato
C	1.74103000	4.79647000	-1.31441000	f=formato
O	-0.25030000	-1.83921000	1.74103000	f=formato
C	-1.31441000	4.79647000	1.74103000	f=formato
O	-0.25030000	1.74103000	5.32127000	f=formato
C	-1.31441000	1.74103000	4.79647000	f=formato
H	1.74103000	-2.07809000	-2.07809000	f=formato
H	5.56015000	-2.07809000	1.74103000	f=formato
H	1.74103000	-2.07809000	5.56015000	f=formato
H	-2.07809000	-2.07809000	1.74103000	f=formato
O	-0.25030000	1.74103000	-1.83921000	f=formato
C	1.74103000	-1.31441000	-1.31441000	f=formato
O	1.74103000	5.32127000	3.73236000	f=formato
O	5.32127000	3.73236000	1.74103000	f=formato
H	1.74103000	5.56015000	5.56015000	f=formato
H	-2.07809000	1.74103000	5.56015000	f=formato
H	-2.07809000	5.56015000	1.74103000	f=formato
H	1.74103000	5.56015000	-2.07809000	f=formato
H	5.56015000	1.74103000	-2.07809000	f=formato
H	5.56015000	5.56015000	1.74103000	f=formato
H	5.56015000	1.74103000	5.56015000	f=formato
H	-2.07809000	1.74103000	-2.07809000	f=formato
O	-1.83921000	3.73236000	1.74103000	f=formato
C	4.79647000	4.79647000	1.74103000	f=formato
O	1.74103000	-1.83921000	-0.25030000	f=formato
O	0.57735000	2.90471000	2.90471000	f=Zr6
O	2.90471000	2.90471000	2.90471000	f=Zr6
H	3.48206000	3.48206000	0.00000000	f=Zr6
O	2.90471000	0.57735000	0.57735000	f=Zr6
H	3.48206000	0.00000000	3.48206000	f=Zr6
O	0.57735000	2.90471000	0.57735000	f=Zr6
H	0.00000000	3.48206000	3.48206000	f=Zr6
O	0.57735000	0.57735000	2.90471000	f=Zr6
H	0.00000000	3.48206000	0.00000000	f=Zr6
H	0.00000000	0.00000000	0.00000000	f=Zr6
H	3.48206000	0.00000000	0.00000000	f=Zr6
Zr	1.74103000	4.23849000	1.74103000	f=Zr6
Zr	1.74103000	1.74103000	-0.75643000	f=Zr6
Zr	1.74103000	-0.75643000	1.74103000	f=Zr6
Zr	1.74103000	1.74103000	4.23849000	f=Zr6
Zr	4.23849000	1.74103000	1.74103000	f=Zr6
Zr	-0.75643000	1.74103000	1.74103000	f=Zr6
O	0.57735000	0.57735000	0.57735000	f=Zr6
O	2.90471000	2.90471000	0.57735000	f=Zr6
O	2.90471000	0.57735000	2.90471000	f=Zr6

Zr, 7 protons

O	3.73236000	-1.83921000	1.74103000	f=formato
O	-1.83921000	-0.25030000	1.74103000	f=formato
O	1.74103000	3.73236000	-1.83921000	f=formato

O	-1.83921000	1.74103000	-0.25030000	f=formato
O	-1.83921000	1.74103000	3.73236000	f=formato
O	1.74103000	3.73236000	5.32127000	f=formato
O	5.32127000	1.74103000	-0.25030000	f=formato
O	1.74103000	-0.25030000	5.32127000	f=formato
O	1.74103000	-0.25030000	-1.83921000	f=formato
O	5.32127000	1.74103000	3.73236000	f=formato
C	4.79647000	1.74103000	4.79647000	f=formato
O	3.73236000	5.32127000	1.74103000	f=formato
O	1.74103000	5.32127000	-0.25030000	f=formato
C	4.79647000	1.74103000	-1.31441000	f=formato
O	1.74103000	-1.83921000	3.73236000	f=formato
O	5.32127000	-0.25030000	1.74103000	f=formato
C	1.74103000	4.79647000	4.79647000	f=formato
C	1.74103000	-1.31441000	4.79647000	f=formato
O	-0.25030000	5.32127000	1.74103000	f=formato
C	4.79647000	-1.31441000	1.74103000	f=formato
O	3.73236000	1.74103000	-1.83921000	f=formato
C	-1.31441000	1.74103000	-1.31441000	f=formato
O	3.73236000	1.74103000	5.32127000	f=formato
C	-1.31441000	-1.31441000	1.74103000	f=formato
C	1.74103000	4.79647000	-1.31441000	f=formato
O	-0.25030000	-1.83921000	1.74103000	f=formato
C	-1.31441000	4.79647000	1.74103000	f=formato
O	-0.25030000	1.74103000	5.32127000	f=formato
C	-1.31441000	1.74103000	4.79647000	f=formato
H	1.74103000	-2.07809000	-2.07809000	f=formato
H	5.56015000	-2.07809000	1.74103000	f=formato
H	1.74103000	-2.07809000	5.56015000	f=formato
H	-2.07809000	-2.07809000	1.74103000	f=formato
O	-0.25030000	1.74103000	-1.83921000	f=formato
C	1.74103000	-1.31441000	-1.31441000	f=formato
O	1.74103000	5.32127000	3.73236000	f=formato
O	5.32127000	3.73236000	1.74103000	f=formato
H	1.74103000	5.56015000	5.56015000	f=formato
H	-2.07809000	1.74103000	5.56015000	f=formato
H	-2.07809000	5.56015000	1.74103000	f=formato
H	1.74103000	5.56015000	-2.07809000	f=formato
H	5.56015000	1.74103000	-2.07809000	f=formato
H	5.56015000	5.56015000	1.74103000	f=formato
H	5.56015000	1.74103000	5.56015000	f=formato
H	-2.07809000	1.74103000	-2.07809000	f=formato
O	-1.83921000	3.73236000	1.74103000	f=formato
C	4.79647000	4.79647000	1.74103000	f=formato
O	1.74103000	-1.83921000	-0.25030000	f=formato
O	0.57735000	2.90471000	2.90471000	f=Zr7
O	2.90471000	2.90471000	2.90471000	f=Zr7
H	3.48206000	3.48206000	0.00000000	f=Zr7
O	2.90471000	0.57735000	0.57735000	f=Zr7
H	3.48206000	0.00000000	3.48206000	f=Zr7
O	0.57735000	2.90471000	0.57735000	f=Zr7
H	0.00000000	3.48206000	3.48206000	f=Zr7
O	0.57735000	0.57735000	2.90471000	f=Zr7
H	0.00000000	3.48206000	0.00000000	f=Zr7
H	0.00000000	0.00000000	0.00000000	f=Zr7
H	3.48206000	0.00000000	0.00000000	f=Zr7
H	3.48206000	3.48206000	3.48206000	f=Zr7
Zr	1.74103000	4.23849000	1.74103000	f=Zr7
Zr	1.74103000	1.74103000	-0.75643000	f=Zr7
Zr	1.74103000	-0.75643000	1.74103000	f=Zr7

Zr	1.74103000	1.74103000	4.23849000	f=Zr7
Zr	4.23849000	1.74103000	1.74103000	f=Zr7
Zr	-0.75643000	1.74103000	1.74103000	f=Zr7
O	0.57735000	0.57735000	0.57735000	f=Zr7
O	2.90471000	2.90471000	0.57735000	f=Zr7
O	2.90471000	0.57735000	2.90471000	f=Zr7

Zr, 8 protons

O	3.73236000	-1.83921000	1.74103000	f=formato
O	-1.83921000	-0.25030000	1.74103000	f=formato
O	1.74103000	3.73236000	-1.83921000	f=formato
O	-1.83921000	1.74103000	-0.25030000	f=formato
O	-1.83921000	1.74103000	3.73236000	f=formato
O	1.74103000	3.73236000	5.32127000	f=formato
O	5.32127000	1.74103000	-0.25030000	f=formato
O	1.74103000	-0.25030000	5.32127000	f=formato
O	1.74103000	-0.25030000	-1.83921000	f=formato
O	5.32127000	1.74103000	3.73236000	f=formato
C	4.79647000	1.74103000	4.79647000	f=formato
O	3.73236000	5.32127000	1.74103000	f=formato
O	1.74103000	5.32127000	-0.25030000	f=formato
C	4.79647000	1.74103000	-1.31441000	f=formato
O	1.74103000	-1.83921000	3.73236000	f=formato
O	5.32127000	-0.25030000	1.74103000	f=formato
C	1.74103000	4.79647000	4.79647000	f=formato
C	1.74103000	-1.31441000	4.79647000	f=formato
O	-0.25030000	5.32127000	1.74103000	f=formato
C	4.79647000	-1.31441000	1.74103000	f=formato
O	3.73236000	1.74103000	-1.83921000	f=formato
C	-1.31441000	1.74103000	-1.31441000	f=formato
O	3.73236000	1.74103000	5.32127000	f=formato
C	-1.31441000	-1.31441000	1.74103000	f=formato
C	1.74103000	4.79647000	-1.31441000	f=formato
O	-0.25030000	-1.83921000	1.74103000	f=formato
C	-1.31441000	4.79647000	1.74103000	f=formato
O	-0.25030000	1.74103000	5.32127000	f=formato
C	-1.31441000	1.74103000	4.79647000	f=formato
H	1.74103000	-2.07809000	-2.07809000	f=formato
H	5.56015000	-2.07809000	1.74103000	f=formato
H	1.74103000	-2.07809000	5.56015000	f=formato
H	-2.07809000	-2.07809000	1.74103000	f=formato
O	-0.25030000	1.74103000	-1.83921000	f=formato
C	1.74103000	-1.31441000	-1.31441000	f=formato
O	1.74103000	5.32127000	3.73236000	f=formato
O	5.32127000	3.73236000	1.74103000	f=formato
H	1.74103000	5.56015000	5.56015000	f=formato
H	-2.07809000	1.74103000	5.56015000	f=formato
H	-2.07809000	5.56015000	1.74103000	f=formato
H	1.74103000	5.56015000	-2.07809000	f=formato
H	5.56015000	1.74103000	-2.07809000	f=formato
H	5.56015000	5.56015000	1.74103000	f=formato
H	5.56015000	1.74103000	5.56015000	f=formato
H	-2.07809000	1.74103000	-2.07809000	f=formato
O	-1.83921000	3.73236000	1.74103000	f=formato
C	4.79647000	4.79647000	1.74103000	f=formato
O	1.74103000	-1.83921000	-0.25030000	f=formato
O	0.57735000	2.90471000	2.90471000	f=Zr8
O	2.90471000	2.90471000	2.90471000	f=Zr8
H	3.48206000	3.48206000	0.00000000	f=Zr8

O	2.90471000	0.57735000	0.57735000	f=Zr8
H	3.48206000	0.00000000	3.48206000	f=Zr8
O	0.57735000	2.90471000	0.57735000	f=Zr8
H	0.00000000	3.48206000	3.48206000	f=Zr8
O	0.57735000	0.57735000	2.90471000	f=Zr8
H	0.00000000	3.48206000	0.00000000	f=Zr8
H	0.00000000	0.00000000	0.00000000	f=Zr8
H	3.48206000	0.00000000	0.00000000	f=Zr8
H	3.48206000	3.48206000	3.48206000	f=Zr8
H	0.00000000	0.00000000	3.48206000	f=Zr8
Zr	1.74103000	4.23849000	1.74103000	f=Zr8
Zr	1.74103000	1.74103000	-0.75643000	f=Zr8
Zr	1.74103000	-0.75643000	1.74103000	f=Zr8
Zr	1.74103000	1.74103000	4.23849000	f=Zr8
Zr	4.23849000	1.74103000	1.74103000	f=Zr8
Zr	-0.75643000	1.74103000	1.74103000	f=Zr8
O	0.57735000	0.57735000	0.57735000	f=Zr8
O	2.90471000	2.90471000	0.57735000	f=Zr8
O	2.90471000	0.57735000	2.90471000	f=Zr8

Hf, 0 protons

O	3.73236000	-1.83921000	1.74103000	f=formato
O	-1.83921000	-0.25030000	1.74103000	f=formato
O	1.74103000	3.73236000	-1.83921000	f=formato
O	-1.83921000	1.74103000	-0.25030000	f=formato
O	-1.83921000	1.74103000	3.73236000	f=formato
O	1.74103000	3.73236000	5.32127000	f=formato
O	5.32127000	1.74103000	-0.25030000	f=formato
O	1.74103000	-0.25030000	5.32127000	f=formato
O	1.74103000	-0.25030000	-1.83921000	f=formato
O	5.32127000	1.74103000	3.73236000	f=formato
C	4.79647000	1.74103000	4.79647000	f=formato
O	3.73236000	5.32127000	1.74103000	f=formato
O	1.74103000	5.32127000	-0.25030000	f=formato
C	4.79647000	1.74103000	-1.31441000	f=formato
O	1.74103000	-1.83921000	3.73236000	f=formato
O	5.32127000	-0.25030000	1.74103000	f=formato
C	1.74103000	4.79647000	4.79647000	f=formato
C	1.74103000	-1.31441000	4.79647000	f=formato
O	-0.25030000	5.32127000	1.74103000	f=formato
C	4.79647000	-1.31441000	1.74103000	f=formato
O	3.73236000	1.74103000	-1.83921000	f=formato
C	-1.31441000	1.74103000	-1.31441000	f=formato
O	3.73236000	1.74103000	5.32127000	f=formato
C	-1.31441000	-1.31441000	1.74103000	f=formato
C	1.74103000	4.79647000	-1.31441000	f=formato
O	-0.25030000	-1.83921000	1.74103000	f=formato
C	-1.31441000	4.79647000	1.74103000	f=formato
O	-0.25030000	1.74103000	5.32127000	f=formato
C	-1.31441000	1.74103000	4.79647000	f=formato
H	1.74103000	-2.07809000	-2.07809000	f=formato
H	5.56015000	-2.07809000	1.74103000	f=formato
H	1.74103000	-2.07809000	5.56015000	f=formato
H	-2.07809000	-2.07809000	1.74103000	f=formato
O	-0.25030000	1.74103000	-1.83921000	f=formato
C	1.74103000	-1.31441000	-1.31441000	f=formato
O	1.74103000	5.32127000	3.73236000	f=formato
O	5.32127000	3.73236000	1.74103000	f=formato
H	1.74103000	5.56015000	5.56015000	f=formato

H	-2.07809000	1.74103000	5.56015000	f=formato
H	-2.07809000	5.56015000	1.74103000	f=formato
H	1.74103000	5.56015000	-2.07809000	f=formato
H	5.56015000	1.74103000	-2.07809000	f=formato
H	5.56015000	5.56015000	1.74103000	f=formato
H	5.56015000	1.74103000	5.56015000	f=formato
H	-2.07809000	1.74103000	-2.07809000	f=formato
O	-1.83921000	3.73236000	1.74103000	f=formato
C	4.79647000	4.79647000	1.74103000	f=formato
O	1.74103000	-1.83921000	-0.25030000	f=formato
O	0.57735000	2.90471000	2.90471000	f=Hf0
O	2.90471000	2.90471000	2.90471000	f=Hf0
O	2.90471000	0.57735000	0.57735000	f=Hf0
O	0.57735000	2.90471000	0.57735000	f=Hf0
O	0.57735000	0.57735000	2.90471000	f=Hf0
Hf	1.74103000	4.23849000	1.74103000	f=Hf0
Hf	1.74103000	1.74103000	-0.75643000	f=Hf0
Hf	1.74103000	-0.75643000	1.74103000	f=Hf0
Hf	1.74103000	1.74103000	4.23849000	f=Hf0
Hf	4.23849000	1.74103000	1.74103000	f=Hf0
Hf	-0.75643000	1.74103000	1.74103000	f=Hf0
O	0.57735000	0.57735000	0.57735000	f=Hf0
O	2.90471000	2.90471000	0.57735000	f=Hf0
O	2.90471000	0.57735000	2.90471000	f=Hf0

Hf, 1 protons

O	3.73236000	-1.83921000	1.74103000	f=formato
O	-1.83921000	-0.25030000	1.74103000	f=formato
O	1.74103000	3.73236000	-1.83921000	f=formato
O	-1.83921000	1.74103000	-0.25030000	f=formato
O	-1.83921000	1.74103000	3.73236000	f=formato
O	1.74103000	3.73236000	5.32127000	f=formato
O	5.32127000	1.74103000	-0.25030000	f=formato
O	1.74103000	-0.25030000	5.32127000	f=formato
O	1.74103000	-0.25030000	-1.83921000	f=formato
O	5.32127000	1.74103000	3.73236000	f=formato
C	4.79647000	1.74103000	4.79647000	f=formato
O	3.73236000	5.32127000	1.74103000	f=formato
O	1.74103000	5.32127000	-0.25030000	f=formato
C	4.79647000	1.74103000	-1.31441000	f=formato
O	1.74103000	-1.83921000	3.73236000	f=formato
O	5.32127000	-0.25030000	1.74103000	f=formato
C	1.74103000	4.79647000	4.79647000	f=formato
C	1.74103000	-1.31441000	4.79647000	f=formato
O	-0.25030000	5.32127000	1.74103000	f=formato
C	4.79647000	-1.31441000	1.74103000	f=formato
O	3.73236000	1.74103000	-1.83921000	f=formato
C	-1.31441000	1.74103000	-1.31441000	f=formato
O	3.73236000	1.74103000	5.32127000	f=formato
C	-1.31441000	-1.31441000	1.74103000	f=formato
C	1.74103000	4.79647000	-1.31441000	f=formato
O	-0.25030000	-1.83921000	1.74103000	f=formato
C	-1.31441000	4.79647000	1.74103000	f=formato
O	-0.25030000	1.74103000	5.32127000	f=formato
C	-1.31441000	1.74103000	4.79647000	f=formato
H	1.74103000	-2.07809000	-2.07809000	f=formato
H	5.56015000	-2.07809000	1.74103000	f=formato
H	1.74103000	-2.07809000	5.56015000	f=formato
H	-2.07809000	-2.07809000	1.74103000	f=formato

O	-0.25030000	1.74103000	-1.83921000	f=formato
C	1.74103000	-1.31441000	-1.31441000	f=formato
O	1.74103000	5.32127000	3.73236000	f=formato
O	5.32127000	3.73236000	1.74103000	f=formato
H	1.74103000	5.56015000	5.56015000	f=formato
H	-2.07809000	1.74103000	5.56015000	f=formato
H	-2.07809000	5.56015000	1.74103000	f=formato
H	1.74103000	5.56015000	-2.07809000	f=formato
H	5.56015000	1.74103000	-2.07809000	f=formato
H	5.56015000	5.56015000	1.74103000	f=formato
H	5.56015000	1.74103000	5.56015000	f=formato
H	-2.07809000	1.74103000	-2.07809000	f=formato
O	-1.83921000	3.73236000	1.74103000	f=formato
C	4.79647000	4.79647000	1.74103000	f=formato
O	1.74103000	-1.83921000	-0.25030000	f=formato
O	0.57735000	2.90471000	2.90471000	f=Hf1
O	2.90471000	2.90471000	2.90471000	f=Hf1
H	3.48206000	0.00000000	0.00000000	f=Hf1
O	2.90471000	0.57735000	0.57735000	f=Hf1
O	0.57735000	2.90471000	0.57735000	f=Hf1
O	0.57735000	0.57735000	2.90471000	f=Hf1
Hf	1.74103000	4.23849000	1.74103000	f=Hf1
Hf	1.74103000	1.74103000	-0.75643000	f=Hf1
Hf	1.74103000	-0.75643000	1.74103000	f=Hf1
Hf	1.74103000	1.74103000	4.23849000	f=Hf1
Hf	4.23849000	1.74103000	1.74103000	f=Hf1
Hf	-0.75643000	1.74103000	1.74103000	f=Hf1
O	0.57735000	0.57735000	0.57735000	f=Hf1
O	2.90471000	2.90471000	0.57735000	f=Hf1
O	2.90471000	0.57735000	2.90471000	f=Hf1

Hf, 2 protons

O	3.73236000	-1.83921000	1.74103000	f=formato
O	-1.83921000	-0.25030000	1.74103000	f=formato
O	1.74103000	3.73236000	-1.83921000	f=formato
O	-1.83921000	1.74103000	-0.25030000	f=formato
O	-1.83921000	1.74103000	3.73236000	f=formato
O	1.74103000	3.73236000	5.32127000	f=formato
O	5.32127000	1.74103000	-0.25030000	f=formato
O	1.74103000	-0.25030000	5.32127000	f=formato
O	1.74103000	-0.25030000	-1.83921000	f=formato
O	5.32127000	1.74103000	3.73236000	f=formato
C	4.79647000	1.74103000	4.79647000	f=formato
O	3.73236000	5.32127000	1.74103000	f=formato
O	1.74103000	5.32127000	-0.25030000	f=formato
C	4.79647000	1.74103000	-1.31441000	f=formato
O	1.74103000	-1.83921000	3.73236000	f=formato
O	5.32127000	-0.25030000	1.74103000	f=formato
C	1.74103000	4.79647000	4.79647000	f=formato
C	1.74103000	-1.31441000	4.79647000	f=formato
O	-0.25030000	5.32127000	1.74103000	f=formato
C	4.79647000	-1.31441000	1.74103000	f=formato
O	3.73236000	1.74103000	-1.83921000	f=formato
C	-1.31441000	1.74103000	-1.31441000	f=formato
O	3.73236000	1.74103000	5.32127000	f=formato
C	-1.31441000	-1.31441000	1.74103000	f=formato
C	1.74103000	4.79647000	-1.31441000	f=formato
O	-0.25030000	-1.83921000	1.74103000	f=formato
C	-1.31441000	4.79647000	1.74103000	f=formato
O	-0.25030000	1.74103000	5.32127000	f=formato

C	-1.31441000	1.74103000	4.79647000	f=formato
H	1.74103000	-2.07809000	-2.07809000	f=formato
H	5.56015000	-2.07809000	1.74103000	f=formato
H	1.74103000	-2.07809000	5.56015000	f=formato
H	-2.07809000	-2.07809000	1.74103000	f=formato
O	-0.25030000	1.74103000	-1.83921000	f=formato
C	1.74103000	-1.31441000	-1.31441000	f=formato
O	1.74103000	5.32127000	3.73236000	f=formato
O	5.32127000	3.73236000	1.74103000	f=formato
H	1.74103000	5.56015000	5.56015000	f=formato
H	-2.07809000	1.74103000	5.56015000	f=formato
H	-2.07809000	5.56015000	1.74103000	f=formato
H	1.74103000	5.56015000	-2.07809000	f=formato
H	5.56015000	1.74103000	-2.07809000	f=formato
H	5.56015000	5.56015000	1.74103000	f=formato
H	5.56015000	1.74103000	5.56015000	f=formato
H	-2.07809000	1.74103000	-2.07809000	f=formato
O	-1.83921000	3.73236000	1.74103000	f=formato
C	4.79647000	4.79647000	1.74103000	f=formato
O	1.74103000	-1.83921000	-0.25030000	f=formato
O	0.57735000	2.90471000	2.90471000	f=Hf2
O	2.90471000	2.90471000	2.90471000	f=Hf2
H	3.48206000	0.00000000	0.00000000	f=Hf2
O	2.90471000	0.57735000	0.57735000	f=Hf2
O	0.57735000	2.90471000	0.57735000	f=Hf2
H	0.00000000	3.48206000	3.48206000	f=Hf2
O	0.57735000	0.57735000	2.90471000	f=Hf2
Hf	1.74103000	4.23849000	1.74103000	f=Hf2
Hf	1.74103000	1.74103000	-0.75643000	f=Hf2
Hf	1.74103000	-0.75643000	1.74103000	f=Hf2
Hf	1.74103000	1.74103000	4.23849000	f=Hf2
Hf	4.23849000	1.74103000	1.74103000	f=Hf2
Hf	-0.75643000	1.74103000	1.74103000	f=Hf2
O	0.57735000	0.57735000	0.57735000	f=Hf2
O	2.90471000	2.90471000	0.57735000	f=Hf2
O	2.90471000	0.57735000	2.90471000	f=Hf2

Hf, 3 protons

O	3.73236000	-1.83921000	1.74103000	f=formato
O	-1.83921000	-0.25030000	1.74103000	f=formato
O	1.74103000	3.73236000	-1.83921000	f=formato
O	-1.83921000	1.74103000	-0.25030000	f=formato
O	-1.83921000	1.74103000	3.73236000	f=formato
O	1.74103000	3.73236000	5.32127000	f=formato
O	5.32127000	1.74103000	-0.25030000	f=formato
O	1.74103000	-0.25030000	5.32127000	f=formato
O	1.74103000	-0.25030000	-1.83921000	f=formato
O	5.32127000	1.74103000	3.73236000	f=formato
C	4.79647000	1.74103000	4.79647000	f=formato
O	3.73236000	5.32127000	1.74103000	f=formato
O	1.74103000	5.32127000	-0.25030000	f=formato
C	4.79647000	1.74103000	-1.31441000	f=formato
O	1.74103000	-1.83921000	3.73236000	f=formato
O	5.32127000	-0.25030000	1.74103000	f=formato
C	1.74103000	4.79647000	4.79647000	f=formato
C	1.74103000	-1.31441000	4.79647000	f=formato
O	-0.25030000	5.32127000	1.74103000	f=formato
C	4.79647000	-1.31441000	1.74103000	f=formato
O	3.73236000	1.74103000	-1.83921000	f=formato
C	-1.31441000	1.74103000	-1.31441000	f=formato

O	3.73236000	1.74103000	5.32127000	f=formato
C	-1.31441000	-1.31441000	1.74103000	f=formato
C	1.74103000	4.79647000	-1.31441000	f=formato
O	-0.25030000	-1.83921000	1.74103000	f=formato
C	-1.31441000	4.79647000	1.74103000	f=formato
O	-0.25030000	1.74103000	5.32127000	f=formato
C	-1.31441000	1.74103000	4.79647000	f=formato
H	1.74103000	-2.07809000	-2.07809000	f=formato
H	5.56015000	-2.07809000	1.74103000	f=formato
H	1.74103000	-2.07809000	5.56015000	f=formato
H	-2.07809000	-2.07809000	1.74103000	f=formato
O	-0.25030000	1.74103000	-1.83921000	f=formato
C	1.74103000	-1.31441000	-1.31441000	f=formato
O	1.74103000	5.32127000	3.73236000	f=formato
O	5.32127000	3.73236000	1.74103000	f=formato
H	1.74103000	5.56015000	5.56015000	f=formato
H	-2.07809000	1.74103000	5.56015000	f=formato
H	-2.07809000	5.56015000	1.74103000	f=formato
H	1.74103000	5.56015000	-2.07809000	f=formato
H	5.56015000	1.74103000	-2.07809000	f=formato
H	5.56015000	5.56015000	1.74103000	f=formato
H	5.56015000	1.74103000	5.56015000	f=formato
H	-2.07809000	1.74103000	-2.07809000	f=formato
O	-1.83921000	3.73236000	1.74103000	f=formato
C	4.79647000	4.79647000	1.74103000	f=formato
O	1.74103000	-1.83921000	-0.25030000	f=formato
O	0.57735000	2.90471000	2.90471000	f=Hf3
O	2.90471000	2.90471000	2.90471000	f=Hf3
H	3.48206000	3.48206000	0.00000000	f=Hf3
O	2.90471000	0.57735000	0.57735000	f=Hf3
H	3.48206000	0.00000000	3.48206000	f=Hf3
O	0.57735000	2.90471000	0.57735000	f=Hf3
H	0.00000000	3.48206000	3.48206000	f=Hf3
O	0.57735000	0.57735000	2.90471000	f=Hf3
Hf	1.74103000	4.23849000	1.74103000	f=Hf3
Hf	1.74103000	1.74103000	-0.75643000	f=Hf3
Hf	1.74103000	-0.75643000	1.74103000	f=Hf3
Hf	1.74103000	1.74103000	4.23849000	f=Hf3
Hf	4.23849000	1.74103000	1.74103000	f=Hf3
Hf	-0.75643000	1.74103000	1.74103000	f=Hf3
O	0.57735000	0.57735000	0.57735000	f=Hf3
O	2.90471000	2.90471000	0.57735000	f=Hf3
O	2.90471000	0.57735000	2.90471000	f=Hf3

Hf, 4 protons

O	3.73236000	-1.83921000	1.74103000	f=formato
O	-1.83921000	-0.25030000	1.74103000	f=formato
O	1.74103000	3.73236000	-1.83921000	f=formato
O	-1.83921000	1.74103000	-0.25030000	f=formato
O	-1.83921000	1.74103000	3.73236000	f=formato
O	1.74103000	3.73236000	5.32127000	f=formato
O	5.32127000	1.74103000	-0.25030000	f=formato
O	1.74103000	-0.25030000	5.32127000	f=formato
O	1.74103000	-0.25030000	-1.83921000	f=formato
O	5.32127000	1.74103000	3.73236000	f=formato
C	4.79647000	1.74103000	4.79647000	f=formato
O	3.73236000	5.32127000	1.74103000	f=formato
O	1.74103000	5.32127000	-0.25030000	f=formato
C	4.79647000	1.74103000	-1.31441000	f=formato
O	1.74103000	-1.83921000	3.73236000	f=formato

O	5.32127000	-0.25030000	1.74103000	f=formato
C	1.74103000	4.79647000	4.79647000	f=formato
C	1.74103000	-1.31441000	4.79647000	f=formato
O	-0.25030000	5.32127000	1.74103000	f=formato
C	4.79647000	-1.31441000	1.74103000	f=formato
O	3.73236000	1.74103000	-1.83921000	f=formato
C	-1.31441000	1.74103000	-1.31441000	f=formato
O	3.73236000	1.74103000	5.32127000	f=formato
C	-1.31441000	-1.31441000	1.74103000	f=formato
C	1.74103000	4.79647000	-1.31441000	f=formato
O	-0.25030000	-1.83921000	1.74103000	f=formato
C	-1.31441000	4.79647000	1.74103000	f=formato
O	-0.25030000	1.74103000	5.32127000	f=formato
C	-1.31441000	1.74103000	4.79647000	f=formato
H	1.74103000	-2.07809000	-2.07809000	f=formato
H	5.56015000	-2.07809000	1.74103000	f=formato
H	1.74103000	-2.07809000	5.56015000	f=formato
H	-2.07809000	-2.07809000	1.74103000	f=formato
O	-0.25030000	1.74103000	-1.83921000	f=formato
C	1.74103000	-1.31441000	-1.31441000	f=formato
O	1.74103000	5.32127000	3.73236000	f=formato
O	5.32127000	3.73236000	1.74103000	f=formato
H	1.74103000	5.56015000	5.56015000	f=formato
H	-2.07809000	1.74103000	5.56015000	f=formato
H	-2.07809000	5.56015000	1.74103000	f=formato
H	1.74103000	5.56015000	-2.07809000	f=formato
H	5.56015000	1.74103000	-2.07809000	f=formato
H	5.56015000	5.56015000	1.74103000	f=formato
H	5.56015000	1.74103000	5.56015000	f=formato
H	-2.07809000	1.74103000	-2.07809000	f=formato
O	-1.83921000	3.73236000	1.74103000	f=formato
C	4.79647000	4.79647000	1.74103000	f=formato
O	1.74103000	-1.83921000	-0.25030000	f=formato
O	0.57735000	2.90471000	2.90471000	f=Hf4
O	2.90471000	2.90471000	2.90471000	f=Hf4
H	3.48206000	3.48206000	0.00000000	f=Hf4
O	2.90471000	0.57735000	0.57735000	f=Hf4
H	3.48206000	0.00000000	3.48206000	f=Hf4
O	0.57735000	2.90471000	0.57735000	f=Hf4
H	0.00000000	3.48206000	3.48206000	f=Hf4
O	0.57735000	0.57735000	2.90471000	f=Hf4
H	0.00000000	0.00000000	0.00000000	f=Hf4
Hf	1.74103000	4.23849000	1.74103000	f=Hf4
Hf	1.74103000	1.74103000	-0.75643000	f=Hf4
Hf	1.74103000	-0.75643000	1.74103000	f=Hf4
Hf	1.74103000	1.74103000	4.23849000	f=Hf4
Hf	4.23849000	1.74103000	1.74103000	f=Hf4
Hf	-0.75643000	1.74103000	1.74103000	f=Hf4
O	0.57735000	0.57735000	0.57735000	f=Hf4
O	2.90471000	2.90471000	0.57735000	f=Hf4
O	2.90471000	0.57735000	2.90471000	f=Hf4

Hf, 5 protons

O	3.73236000	-1.83921000	1.74103000	f=formato
O	-1.83921000	-0.25030000	1.74103000	f=formato
O	1.74103000	3.73236000	-1.83921000	f=formato
O	-1.83921000	1.74103000	-0.25030000	f=formato
O	-1.83921000	1.74103000	3.73236000	f=formato
O	1.74103000	3.73236000	5.32127000	f=formato
O	5.32127000	1.74103000	-0.25030000	f=formato

O	1.74103000	-0.25030000	5.32127000	f=formato
O	1.74103000	-0.25030000	-1.83921000	f=formato
O	5.32127000	1.74103000	3.73236000	f=formato
C	4.79647000	1.74103000	4.79647000	f=formato
O	3.73236000	5.32127000	1.74103000	f=formato
O	1.74103000	5.32127000	-0.25030000	f=formato
C	4.79647000	1.74103000	-1.31441000	f=formato
O	1.74103000	-1.83921000	3.73236000	f=formato
O	5.32127000	-0.25030000	1.74103000	f=formato
C	1.74103000	4.79647000	4.79647000	f=formato
C	1.74103000	-1.31441000	4.79647000	f=formato
O	-0.25030000	5.32127000	1.74103000	f=formato
C	4.79647000	-1.31441000	1.74103000	f=formato
O	3.73236000	1.74103000	-1.83921000	f=formato
C	-1.31441000	1.74103000	-1.31441000	f=formato
O	3.73236000	1.74103000	5.32127000	f=formato
C	-1.31441000	-1.31441000	1.74103000	f=formato
C	1.74103000	4.79647000	-1.31441000	f=formato
O	-0.25030000	-1.83921000	1.74103000	f=formato
C	-1.31441000	4.79647000	1.74103000	f=formato
O	-0.25030000	1.74103000	5.32127000	f=formato
C	-1.31441000	1.74103000	4.79647000	f=formato
H	1.74103000	-2.07809000	-2.07809000	f=formato
H	5.56015000	-2.07809000	1.74103000	f=formato
H	1.74103000	-2.07809000	5.56015000	f=formato
H	-2.07809000	-2.07809000	1.74103000	f=formato
O	-0.25030000	1.74103000	-1.83921000	f=formato
C	1.74103000	-1.31441000	-1.31441000	f=formato
O	1.74103000	5.32127000	3.73236000	f=formato
O	5.32127000	3.73236000	1.74103000	f=formato
H	1.74103000	5.56015000	5.56015000	f=formato
H	-2.07809000	1.74103000	5.56015000	f=formato
H	-2.07809000	5.56015000	1.74103000	f=formato
H	1.74103000	5.56015000	-2.07809000	f=formato
H	5.56015000	1.74103000	-2.07809000	f=formato
H	5.56015000	5.56015000	1.74103000	f=formato
H	5.56015000	1.74103000	5.56015000	f=formato
H	-2.07809000	1.74103000	-2.07809000	f=formato
O	-1.83921000	3.73236000	1.74103000	f=formato
C	4.79647000	4.79647000	1.74103000	f=formato
O	1.74103000	-1.83921000	-0.25030000	f=formato
O	0.57735000	2.90471000	2.90471000	f=Hf5
O	2.90471000	2.90471000	2.90471000	f=Hf5
H	3.48206000	3.48206000	0.00000000	f=Hf5
O	2.90471000	0.57735000	0.57735000	f=Hf5
H	3.48206000	0.00000000	3.48206000	f=Hf5
O	0.57735000	2.90471000	0.57735000	f=Hf5
H	0.00000000	3.48206000	3.48206000	f=Hf5
O	0.57735000	0.57735000	2.90471000	f=Hf5
H	0.00000000	0.00000000	0.00000000	f=Hf5
H	3.48206000	3.48206000	3.48206000	f=Hf5
Hf	1.74103000	4.23849000	1.74103000	f=Hf5
Hf	1.74103000	1.74103000	-0.75643000	f=Hf5
Hf	1.74103000	-0.75643000	1.74103000	f=Hf5
Hf	1.74103000	1.74103000	4.23849000	f=Hf5
Hf	4.23849000	1.74103000	1.74103000	f=Hf5
Hf	-0.75643000	1.74103000	1.74103000	f=Hf5
O	0.57735000	0.57735000	0.57735000	f=Hf5
O	2.90471000	2.90471000	0.57735000	f=Hf5
O	2.90471000	0.57735000	2.90471000	f=Hf5

Hf, 6 protons

O	3.73236000	-1.83921000	1.74103000	f=formato
O	-1.83921000	-0.25030000	1.74103000	f=formato
O	1.74103000	3.73236000	-1.83921000	f=formato
O	-1.83921000	1.74103000	-0.25030000	f=formato
O	-1.83921000	1.74103000	3.73236000	f=formato
O	1.74103000	3.73236000	5.32127000	f=formato
O	5.32127000	1.74103000	-0.25030000	f=formato
O	1.74103000	-0.25030000	5.32127000	f=formato
O	1.74103000	-0.25030000	-1.83921000	f=formato
O	5.32127000	1.74103000	3.73236000	f=formato
C	4.79647000	1.74103000	4.79647000	f=formato
O	3.73236000	5.32127000	1.74103000	f=formato
O	1.74103000	5.32127000	-0.25030000	f=formato
C	4.79647000	1.74103000	-1.31441000	f=formato
O	1.74103000	-1.83921000	3.73236000	f=formato
O	5.32127000	-0.25030000	1.74103000	f=formato
C	1.74103000	4.79647000	4.79647000	f=formato
C	1.74103000	-1.31441000	4.79647000	f=formato
O	-0.25030000	5.32127000	1.74103000	f=formato
C	4.79647000	-1.31441000	1.74103000	f=formato
O	3.73236000	1.74103000	-1.83921000	f=formato
C	-1.31441000	1.74103000	-1.31441000	f=formato
O	3.73236000	1.74103000	5.32127000	f=formato
C	-1.31441000	-1.31441000	1.74103000	f=formato
C	1.74103000	4.79647000	-1.31441000	f=formato
O	-0.25030000	-1.83921000	1.74103000	f=formato
C	-1.31441000	4.79647000	1.74103000	f=formato
O	-0.25030000	1.74103000	5.32127000	f=formato
C	-1.31441000	1.74103000	4.79647000	f=formato
H	1.74103000	-2.07809000	-2.07809000	f=formato
H	5.56015000	-2.07809000	1.74103000	f=formato
H	1.74103000	-2.07809000	5.56015000	f=formato
H	-2.07809000	-2.07809000	1.74103000	f=formato
O	-0.25030000	1.74103000	-1.83921000	f=formato
C	1.74103000	-1.31441000	-1.31441000	f=formato
O	1.74103000	5.32127000	3.73236000	f=formato
O	5.32127000	3.73236000	1.74103000	f=formato
H	1.74103000	5.56015000	5.56015000	f=formato
H	-2.07809000	1.74103000	5.56015000	f=formato
H	-2.07809000	5.56015000	1.74103000	f=formato
H	1.74103000	5.56015000	-2.07809000	f=formato
H	5.56015000	1.74103000	-2.07809000	f=formato
H	5.56015000	5.56015000	1.74103000	f=formato
H	5.56015000	1.74103000	5.56015000	f=formato
H	-2.07809000	1.74103000	-2.07809000	f=formato
O	-1.83921000	3.73236000	1.74103000	f=formato
C	4.79647000	4.79647000	1.74103000	f=formato
O	1.74103000	-1.83921000	-0.25030000	f=formato
O	0.57735000	2.90471000	2.90471000	f=Hf6
O	2.90471000	2.90471000	2.90471000	f=Hf6
H	3.48206000	3.48206000	0.00000000	f=Hf6
O	2.90471000	0.57735000	0.57735000	f=Hf6
H	3.48206000	0.00000000	3.48206000	f=Hf6
O	0.57735000	2.90471000	0.57735000	f=Hf6
H	0.00000000	3.48206000	3.48206000	f=Hf6
O	0.57735000	0.57735000	2.90471000	f=Hf6
H	0.00000000	3.48206000	0.00000000	f=Hf6
H	0.00000000	0.00000000	0.00000000	f=Hf6

H	3.48206000	3.48206000	3.48206000	f=Hf6
Hf	1.74103000	4.23849000	1.74103000	f=Hf6
Hf	1.74103000	1.74103000	-0.75643000	f=Hf6
Hf	1.74103000	-0.75643000	1.74103000	f=Hf6
Hf	1.74103000	1.74103000	4.23849000	f=Hf6
Hf	4.23849000	1.74103000	1.74103000	f=Hf6
Hf	-0.75643000	1.74103000	1.74103000	f=Hf6
O	0.57735000	0.57735000	0.57735000	f=Hf6
O	2.90471000	2.90471000	0.57735000	f=Hf6
O	2.90471000	0.57735000	2.90471000	f=Hf6

Hf, 7 protons

O	3.73236000	-1.83921000	1.74103000	f=formato
O	-1.83921000	-0.25030000	1.74103000	f=formato
O	1.74103000	3.73236000	-1.83921000	f=formato
O	-1.83921000	1.74103000	-0.25030000	f=formato
O	-1.83921000	1.74103000	3.73236000	f=formato
O	1.74103000	3.73236000	5.32127000	f=formato
O	5.32127000	1.74103000	-0.25030000	f=formato
O	1.74103000	-0.25030000	5.32127000	f=formato
O	1.74103000	-0.25030000	-1.83921000	f=formato
O	5.32127000	1.74103000	3.73236000	f=formato
C	4.79647000	1.74103000	4.79647000	f=formato
O	3.73236000	5.32127000	1.74103000	f=formato
O	1.74103000	5.32127000	-0.25030000	f=formato
C	4.79647000	1.74103000	-1.31441000	f=formato
O	1.74103000	-1.83921000	3.73236000	f=formato
O	5.32127000	-0.25030000	1.74103000	f=formato
C	1.74103000	4.79647000	4.79647000	f=formato
C	1.74103000	-1.31441000	4.79647000	f=formato
O	-0.25030000	5.32127000	1.74103000	f=formato
C	4.79647000	-1.31441000	1.74103000	f=formato
O	3.73236000	1.74103000	-1.83921000	f=formato
C	-1.31441000	1.74103000	-1.31441000	f=formato
O	3.73236000	1.74103000	5.32127000	f=formato
C	-1.31441000	-1.31441000	1.74103000	f=formato
C	1.74103000	4.79647000	-1.31441000	f=formato
O	-0.25030000	-1.83921000	1.74103000	f=formato
C	-1.31441000	4.79647000	1.74103000	f=formato
O	-0.25030000	1.74103000	5.32127000	f=formato
C	-1.31441000	1.74103000	4.79647000	f=formato
H	1.74103000	-2.07809000	-2.07809000	f=formato
H	5.56015000	-2.07809000	1.74103000	f=formato
H	1.74103000	-2.07809000	5.56015000	f=formato
H	-2.07809000	-2.07809000	1.74103000	f=formato
O	-0.25030000	1.74103000	-1.83921000	f=formato
C	1.74103000	-1.31441000	-1.31441000	f=formato
O	1.74103000	5.32127000	3.73236000	f=formato
O	5.32127000	3.73236000	1.74103000	f=formato
H	1.74103000	5.56015000	5.56015000	f=formato
H	-2.07809000	1.74103000	5.56015000	f=formato
H	-2.07809000	5.56015000	1.74103000	f=formato
H	1.74103000	5.56015000	-2.07809000	f=formato
H	5.56015000	1.74103000	-2.07809000	f=formato
H	5.56015000	5.56015000	1.74103000	f=formato
H	5.56015000	1.74103000	5.56015000	f=formato
H	-2.07809000	1.74103000	-2.07809000	f=formato
O	-1.83921000	3.73236000	1.74103000	f=formato
C	4.79647000	4.79647000	1.74103000	f=formato
O	1.74103000	-1.83921000	-0.25030000	f=formato

O	0.57735000	2.90471000	2.90471000	f=Hf7
O	2.90471000	2.90471000	2.90471000	f=Hf7
H	3.48206000	3.48206000	0.00000000	f=Hf7
O	2.90471000	0.57735000	0.57735000	f=Hf7
H	3.48206000	0.00000000	3.48206000	f=Hf7
O	0.57735000	2.90471000	0.57735000	f=Hf7
H	0.00000000	3.48206000	3.48206000	f=Hf7
O	0.57735000	0.57735000	2.90471000	f=Hf7
H	0.00000000	3.48206000	0.00000000	f=Hf7
H	0.00000000	0.00000000	0.00000000	f=Hf7
H	3.48206000	0.00000000	0.00000000	f=Hf7
H	3.48206000	3.48206000	3.48206000	f=Hf7
Hf	1.74103000	4.23849000	1.74103000	f=Hf7
Hf	1.74103000	1.74103000	-0.75643000	f=Hf7
Hf	1.74103000	-0.75643000	1.74103000	f=Hf7
Hf	1.74103000	1.74103000	4.23849000	f=Hf7
Hf	4.23849000	1.74103000	1.74103000	f=Hf7
Hf	-0.75643000	1.74103000	1.74103000	f=Hf7
O	0.57735000	0.57735000	0.57735000	f=Hf7
O	2.90471000	2.90471000	0.57735000	f=Hf7
O	2.90471000	0.57735000	2.90471000	f=Hf7

Hf, 8 protons

O	3.73236000	-1.83921000	1.74103000	f=formato
O	-1.83921000	-0.25030000	1.74103000	f=formato
O	1.74103000	3.73236000	-1.83921000	f=formato
O	-1.83921000	1.74103000	-0.25030000	f=formato
O	-1.83921000	1.74103000	3.73236000	f=formato
O	1.74103000	3.73236000	5.32127000	f=formato
O	5.32127000	1.74103000	-0.25030000	f=formato
O	1.74103000	-0.25030000	5.32127000	f=formato
O	1.74103000	-0.25030000	-1.83921000	f=formato
O	5.32127000	1.74103000	3.73236000	f=formato
C	4.79647000	1.74103000	4.79647000	f=formato
O	3.73236000	5.32127000	1.74103000	f=formato
O	1.74103000	5.32127000	-0.25030000	f=formato
C	4.79647000	1.74103000	-1.31441000	f=formato
O	1.74103000	-1.83921000	3.73236000	f=formato
O	5.32127000	-0.25030000	1.74103000	f=formato
C	1.74103000	4.79647000	4.79647000	f=formato
C	1.74103000	-1.31441000	4.79647000	f=formato
O	-0.25030000	5.32127000	1.74103000	f=formato
C	4.79647000	-1.31441000	1.74103000	f=formato
O	3.73236000	1.74103000	-1.83921000	f=formato
C	-1.31441000	1.74103000	-1.31441000	f=formato
O	3.73236000	1.74103000	5.32127000	f=formato
C	-1.31441000	-1.31441000	1.74103000	f=formato
C	1.74103000	4.79647000	-1.31441000	f=formato
O	-0.25030000	-1.83921000	1.74103000	f=formato
C	-1.31441000	4.79647000	1.74103000	f=formato
O	-0.25030000	1.74103000	5.32127000	f=formato
C	-1.31441000	1.74103000	4.79647000	f=formato
H	1.74103000	-2.07809000	-2.07809000	f=formato
H	5.56015000	-2.07809000	1.74103000	f=formato
H	1.74103000	-2.07809000	5.56015000	f=formato
H	-2.07809000	-2.07809000	1.74103000	f=formato
O	-0.25030000	1.74103000	-1.83921000	f=formato
C	1.74103000	-1.31441000	-1.31441000	f=formato
O	1.74103000	5.32127000	3.73236000	f=formato
O	5.32127000	3.73236000	1.74103000	f=formato

H	1.74103000	5.56015000	5.56015000	f=formato
H	-2.07809000	1.74103000	5.56015000	f=formato
H	-2.07809000	5.56015000	1.74103000	f=formato
H	1.74103000	5.56015000	-2.07809000	f=formato
H	5.56015000	1.74103000	-2.07809000	f=formato
H	5.56015000	5.56015000	1.74103000	f=formato
H	5.56015000	1.74103000	5.56015000	f=formato
H	-2.07809000	1.74103000	-2.07809000	f=formato
O	-1.83921000	3.73236000	1.74103000	f=formato
C	4.79647000	4.79647000	1.74103000	f=formato
O	1.74103000	-1.83921000	-0.25030000	f=formato
O	0.57735000	2.90471000	2.90471000	f=Hf8
O	2.90471000	2.90471000	2.90471000	f=Hf8
H	3.48206000	3.48206000	0.00000000	f=Hf8
O	2.90471000	0.57735000	0.57735000	f=Hf8
H	3.48206000	0.00000000	3.48206000	f=Hf8
O	0.57735000	2.90471000	0.57735000	f=Hf8
H	0.00000000	3.48206000	3.48206000	f=Hf8
O	0.57735000	0.57735000	2.90471000	f=Hf8
H	0.00000000	3.48206000	0.00000000	f=Hf8
H	0.00000000	0.00000000	0.00000000	f=Hf8
H	3.48206000	0.00000000	0.00000000	f=Hf8
H	3.48206000	3.48206000	3.48206000	f=Hf8
H	0.00000000	0.00000000	3.48206000	f=Hf8
Hf	1.74103000	4.23849000	1.74103000	f=Hf8
Hf	1.74103000	1.74103000	-0.75643000	f=Hf8
Hf	1.74103000	-0.75643000	1.74103000	f=Hf8
Hf	1.74103000	1.74103000	4.23849000	f=Hf8
Hf	4.23849000	1.74103000	1.74103000	f=Hf8
Hf	-0.75643000	1.74103000	1.74103000	f=Hf8
O	0.57735000	0.57735000	0.57735000	f=Hf8
O	2.90471000	2.90471000	0.57735000	f=Hf8
O	2.90471000	0.57735000	2.90471000	f=Hf8

Supplementary methods

Integral SAXS parameters

The definition of scattering vector q is $q=(4\pi/\lambda)\sin\theta$ (dimension in nm^{-1}), where θ is the Bragg angle that is usually used in XRD. The physical meaning of q is that all electrons in a plane perpendicular to q are in phase with respect to incoming light. The location of a particular electron with respect to an electron in the origin (spherical coordinates (0,0,0)) is defined by r. Therefore the phase of the electron is qr and the outcoming *phase factor* as recorded is given by e^{-iqr}, which is the complex representation that is convenient for Fourier Transformation (FT). The magnitude of q is representative for the size of the scattering entities; the typical scale of the scatterer is of the order $2\pi/q$.
Now, the recorded amplitude is the sum of all recorded waves, which in our case is defined using an *electron density* $\rho(r)$, integrated over all three dimensions:

$$A(q) = \iiint dV \cdot \rho(r) e^{-iqr} \quad \textbf{Eq. S.1}$$

This is a Fourier Integral. Mathematically speaking, we need to square the amplitude by multiplying it by its complex conjugate to yield intensity. Physically speaking, only a *pair* of electrons can be responsible for interference, and therefore the diffraction pattern. The result of both interpretations is the same and yields a six-fold integral over two volume elements and two local spaces, with the distance between the electron pair defined as r_1-r_2. Rather than evaluating this integral, every pair of electrons is defined by one single, fictitious point $r = r_1 - r_2$. This transformation is a FT in itself, and yields a new relation for $I(q)$:

$$I(q) = \iiint dV \langle \rho(r) \rangle^2 e^{-iqr} \quad \textbf{Eq. S.2}$$

Here, the angular brackets denote the auto-correlation, the summarizing of all electron pairs.

An additional advantage to mathematical simplification is that this –new- electron density is averaged over all electrons in the structure, and is highly characteristic for the morphology of the scatterer.
When we take into account the isotropy in space, the problem becomes one-dimensional in r, and Debye formulated an orientationally averaged term for the phase factor.[31]

$$\langle e^{-iqr} \rangle = \frac{\sin(qr)}{qr} \quad \textbf{Eq. S.3}$$

Then, making use of the absence of long-range order in a solute or dispersed system of scatterers, one measures only fluctuation relative to the background:

$$\eta = \rho - \bar{\rho} \quad \textbf{Eq. S.4}$$

For which:

$$\tilde{\eta}^2 = V \cdot \gamma(r) \quad \textbf{Eq. S.5}$$

that has defined an *auto-correlation* by:

$$\gamma(r) = \langle \eta(r_1)\eta(r_2) \rangle, r = r_1 - r_2 \quad \textbf{Eq. S.6}$$

$\gamma(r)$ is therefore the average of the two fluctuations occurring at a distance r (the distance between the electron pair. This auto-correlation function is very distinct for the geometry of

scattering entity. In our case we don't have very well defined crystals and we use the *pair-distance distribution p(r)* instead, which is derived below.
First, one must summarize the simplifications and formulations of Eq. S.3 to Eq. S.6, in which the equation for intensity is made one-dimensional in spherical coordinates, the phase factor is simplified by the Debye equation, and electron density is transformed into an auto-correlation which is a deviation from the background and is directly characteristic for the geometry of the scatterer.
One can now write Eq. S.2 as:

$$I(q) = V\int_0^\infty 4\pi r^2 dr \cdot \gamma(r)\frac{\sin(qr)}{qr} \qquad \textbf{Eq. S.7}$$

This functional describes the shape of the intensity decay obtained in the SAXS experiment. The Inverse Fourier Transform of *I(q)* yields the function for *γ(r)*, which by multiplication with r^2 yields *p(r)*, carrying dimension [nm]:

$$p(r) = \frac{r^2}{V}\frac{1}{2\pi^2}\int_0^\infty q^2 dq \cdot I(q)\frac{\sin(qr)}{qr} \qquad \textbf{Eq. S.8}$$

The *p(r)* function describes the paired-set of all distances between points within an object, and is used here to detect conformational changes in time within the growing MOF particles.

Before continuing, we must first consider that in order to perform FT analysis, a smooth dataset spanning from 0 to ∞ is needed. Experimental SAXS data have a cut-off at the beam stop at a small *q*-value (the value *q* = 0 can't be measured), plus a nonzero background value at large *q*, resulting in infinite values for the Fourier integral.
The first problem can be solved by extrapolating SAXS data to *q* = 0 by using an approximation derived by Guinier in 1939 for single-particle scattering.[32] The derivation is briefly discussed here. Two major criteria – which are not always met in literature – but which are required for the Guinier approximation to be valid are the following: (1) the particles must be well-separated in solution (2) there scatterer must have a centre of symmetry. In other words, the solution in which the MOF crystallises ought to be (at least relatively) dilute, and the MOF crystals and agglomerates should possess an axis of symmetry. The first criterium is met since – even though not all 2-aminoterephthalic acid is dissolved at the start of synthesis – crystallisation occurs from clear solutions. The second criterium also holds for the NH_2-MIL-53(Al) and NH_2-MIL-101(Al) frameworks, which on average, and to reasonable extent, possess a centre of symmetry.
With condition (1), one can focus on the scattering by a single particle, and condition (2) simplifies the phase factor e^{-iqr}, which has now become real for any orientation, and can be replaced by *cos(qr).* Now, Eq. S.1, for the amplitude, becomes:

$$A_1(q) = \Delta\rho \int dV \cdot \cos(qr) \qquad \textbf{Eq. S.9}$$

Where $\Delta\rho=\rho-\rho_0$, ρ_0 being the scattering from solution. This is implied by condition (1) and *Δρ,* which is independent of *r*, replaces *ρ(r)* of Eq. S.1. Now, *cos(qr)* can be expanded via:

$$\cos(qr) = 1 - \frac{(hr)^2}{2} + \cdots \qquad \textbf{Eq. S.10}$$

Which leads to the *Guinier Approximation*

$$I_1(q) = (\Delta n_e)^2 e^{-q^2 R_g^2/3} \qquad \textbf{Eq. S.11}$$

Where $\Delta n_e = (\Delta\rho V)^2$ and R_g is the *Radius of gyration.*

In the data treatment R_g is used to extrapolate data to $q = 0$.
The second problem, the subtraction of the background and infinite integration can be treated using the approximation for the final slope as derived by Porod.[32] For this, a scaled version of the correlation function is defined, such that:

$$\gamma_0(r) = \frac{\gamma(r)}{(\Delta\rho)^2} \qquad \textbf{Eq. S.12}$$

This function was called the *characteristic* by Porod and is analytically derivable for most well-defined, constant morphologies, which opens the door to modeling

This is done to obtain a parameter, $\gamma_0(r)$, which is related to the electronic structure (read: morphology) of the scatterer, and scaled by the electron density difference, which is usually assumed constant. $\gamma_0(r)$ plays an important role in modeling with SAXS, by defining a *correlation length,* but this is not considered in our data treatment, and will not be further considered (the interested reader is referred to the standard works of Glatter & Kratky and Feigin & Svergun). [32,33]

For the *Porod regime* of the decay in SAXS (the higher *q*-values), $\gamma_0(r)$ can be expanded into a power series

$$\gamma_0(r) = 1 - ar + br^2 + cr^3 \dots \qquad \textbf{Eq. S.13}$$

Now, it is possible to define $\gamma_0(r)$ as the volumetic overlap of a particle and a new particle moved by a distance *r*. For small *r*, it is obvious that $\gamma_0(r)$ is determined by the surface of the particle. This leads to the derivation of the fourth power law (see again: Glatter and Kratky), defined as:

$$\frac{S}{V} = \pi \cdot \lim_{q\to\infty} I(q)q^4/Q^{inv} \qquad \textbf{Eq. S.14}$$

Here *S/V* is the Porod surface-to-volume ratio of the scatterer, and Q^{inv} is the Porod *Invariant.* The latter needs consideration, as it is a fundamental parameter in SAXS. Setting $r = 0$ in $\gamma(r)$ leads to:

$$\gamma(0) = \frac{1}{2\pi^2 V}\int_0^\infty q^2 dq \cdot I(q) = \overline{\eta^2} \qquad \textbf{Eq. S.15}$$

The final statement is easily derived from the definition of the auto-correlation (Eq. S.6), for which:

$$\lim_{r\to\infty} \gamma(r) = 0 \qquad \textbf{Eq. S.16}$$

$$\gamma(0) = \overline{\eta^2} \qquad \textbf{Eq. S.17}$$

Eq. S.16 follows from the fact that, at large electron-pair distances, ‘correlation is lost’, i.e. the average local scattering is equal to the scattering of the background. It must be stated that, although here the limit of *r* to infinity is taken, in reality, $\gamma(r)$ goes to zero for very finite values that lie well within colloidal dimensions. Eq. S.17 follows directly from the definition of the correlation function in Eq. S.6. The result is important as it means that the integral factor in Eq.S.15 is only directly proportional to the mean square fluctuation of electron density, meaning that it is fully independent of temperature, crystallinity and/or crystal morphology, and is therefore called the *Porod Invariant* Q^{inv}*:*

$$Q^{inv} = \int_0^\infty q^2 dq \cdot I(q) \qquad \textbf{Eq. S.18}$$

Using this definition, Eq. S.11 and Eq. S.14 are used to extrapolate the pattern obtained by synchrotron SAXS data (figure 3B.1). Here the power law in Eq. S.14 is used to extrapolate the *q*-space to infinity, using $I(q) = x_1q^{-4} + x_2$. Here, x_1 represents $SQ/\pi V$ while x_2 corrects for incomplete subtraction of the background from experimental data. This data treatment yields a smooth dataset without Bragg peaks suitable for the application of eqs 3B.11 and 3B.14. Functions and parameters that can thus be calculated in this time-resolved experiment are: $p(r)$, R_g, V, and S/V. The first and the third are of most importance in this chapter, and therefore leaves us with the definition of V, the *Porod Volume*. Using the condition that for a single scatterer:

$$I_1(0) = (\Delta\rho)^2 \cdot V^2 = (\Delta n_e)^2 \qquad \textbf{Eq. S.19}$$

This is the condition that $I_1(0)$ must be equal to the square of the number of excess electrons presented by the scatterer. From this follows that:

$$Q_1^{inv} = \int_0^\infty q^2 dq \cdot I_1(q) = 2\pi^2 \cdot (\Delta\rho)^2 \cdot V \qquad \textbf{Eq. S.20}$$

Here it was used that for a single particle

$$\overline{\eta^2} = (\Delta\rho)^2 \qquad \textbf{Eq. S.21}$$

Combination of Eq.S.19 with Eq.S.18 yields the expression for the Porod Volume, V, of the scatterer:

$$V = I_1(0)/Q_1^{inv} \cdot 2\pi^2 \qquad \textbf{Eq. S.22}$$

As it is deduced for a single-scatterer, the same condition as for the radius of gyration holds, in the sense that the equation does not hold well for very densely packed systems of scatterers.

When dealing with crystals that do not possess any high aspect ratios, deviation from the fourth power law of Porod might occur when the surface of the scatterer is not smooth. In that case $I(q)$ obeys behaviour of the form q^{6-D_s} with D_s representing the dimensionality of the surface fractal of the scatterer. Power law decay of the form $q^{-\alpha}$ with $\alpha = 4$ indicates a scatterer with a smooth surface. When $\alpha < 4$, one deals with rougher surfaces. As α approaches 3, one deals with surfaces of dominant fractal character.

Supplementary reference

1. Grimme, S., Antony, J., Ehrlich, S. & Krieg, H. A consistent and accurate ab initio parametrization of density functional dispersion correction (DFT-D) for the 94 elements H-Pu. *The Journal of Chemical Physics* **132,** (2010).
